# VAMP3 vesicles support host protein recruitment and *Plasmodium* liver stage development

**DOI:** 10.1128/mbio.01430-26

**Published:** 2026-07-21

**Authors:** Michael E. Chirgwin, Chelsea Gan, Karla P. Godinez-Macias, Kaitlyn Choe, Justine C. Shiau, Jaeson Calla, Dennis E. Kyle, Elizabeth A. Winzeler, Emily R. Derbyshire

**Affiliations:** 1 Department of Chemistry, Duke University 201942, Durham, North Carolina, USA; 2 Department of Pediatrics, School of Medicine, University of California, San Diego 12220, La Jolla, California, USA; 3 Center for Tropical and Emerging Global Diseases, University of Georgia 1355, Athens, Georgia, USA; 4 Doctoral Programme in Health Sciences, Faculty of Medicine, Universidad Nacional Mayor de San Marcos 33209, Lima, Peru; 5 Skaggs School of Pharmacy and Pharmaceutical Sciences, University of California, San Diego 15500, La Jolla, California, USA; 6 Department of Molecular Genetics and Microbiology, Duke University School of Medicine 12277, Durham, North Carolina, USA; Rutgers-New Jersey Medical School, Newark, New Jersey, USA

**Keywords:** *Plasmodium*, liver stage, VAMP3, vesicle fusion, vesicular trafficking

## Abstract

*Plasmodium* remodels the host liver cell as an obligatory step in malaria infection. Although several host proteins are recruited to the parasitophorous vacuole membrane (PVM) after invasion, the mechanisms underlying this acquisition remain unknown. Here, we validated the upregulation and recruitment of the host protein CGA during liver stage *Plasmodium berghei* infection. We then depleted a panel of host trafficking proteins to investigate their role in CGA recruitment to the *P. berghei* PVM. This highlighted the significance of protein exocytosis for host protein acquisition and identified vesicle-associated membrane protein 3 (VAMP3), a SNARE family protein, as critical for CGA recruitment and parasite growth. A phenotypic study revealed that *VAMP3* depletion decreased *P. berghei* survival after invasion, which correlated with enhanced LC3 recruitment. VAMP3 recruitment to the PVM was observed in the rodent-infective *P. berghei* and the human-infective *Plasmodium falciparum,* suggesting host vesicle redirection may be a conserved mechanism during the parasite’s liver stage. Expansion microscopy revealed VAMP3 vesicles near and overlapping with the PVM, suggesting fusion events might occur. This proposal was tested using the chemical probe N-ethylmaleimide (NEM), which prevents SNARE recycling. VAMP3 accumulates on the PVM after NEM treatment, consistent with host vesicle fusion to the PVM. Overall, we uncover factors that facilitate *Plasmodium’s* acquisition of host proteins and provide evidence that the PVM undergoes fusion with host SNARE machinery.

**IMPORTANCE:**

*Plasmodium* remodels the host liver cell as an essential step in malaria infection, yet the mechanisms by which host proteins are acquired at the parasitophorous vacuole membrane (PVM) remain unclear. Here, we show that the host protein CGA is upregulated in the *Plasmodium berghei* liver stage and recruited to the PVM by host exocytosis machinery. Specifically, the SNARE protein vesicle-associated membrane protein 3 (VAMP3) is linked to CGA acquisition and is shown to be recruited to the PVM in both rodent- and human-infective malaria. Functional VAMP3 is critical for *P. berghei* growth and correlates with its survival. Imaging and chemical inhibition studies provide evidence that supports host vesicle fusion with the PVM for the first time. Overall, these findings reveal that host SNARE machinery facilitates *Plasmodium* acquisition of host proteins.

## INTRODUCTION

*Plasmodium* parasites cause malaria, a deadly disease that infected over 280 million people and killed more than 610 thousand in 2024 (1). Infection begins when a *Plasmodium*-infected female *Anopheles* mosquito deposits sporozoites into the skin of a vertebrate host (2–4). After traveling through the bloodstream to the liver, the parasites traverse through several hepatocytes before invasion via invagination of the host plasma membrane (5). Within a hepatocyte, the parasite safely resides in the parasitophorous vacuole (PV), with the PV membrane (PVM) serving as the host–parasite interface (6). In the liver stage (LS), a single parasite will undergo multiple rounds of DNA replication and schizogony, yielding tens of thousands of merozoites. These merozoites travel to the bloodstream, where they cyclically invade and burst red blood cells (RBCs), resulting in the clinical symptoms of malaria (2).

As an obligate intracellular pathogen, *Plasmodium* manipulates the host cell throughout its life cycle for survival. During the *Plasmodium* blood stage, hundreds of effector proteins are secreted to remodel the host erythrocyte (7). During the LS, effector proteins may also be secreted, and infection is associated with significant changes in host gene expression. This has been demonstrated through bulk analyses with *Plasmodium berghei*, a mouse-infective parasite used as a model organism (8–10), and single-cell RNA-seq studies with the human-infective *Plasmodium falciparum* and *Plasmodium vivax,* in addition to *P. berghei* (11–16). From these studies and others, it is known that the parasite downregulates apoptotic factors (P53, TNF-α, and cytochrome c), upregulates pro-survival factors (hepatocyte growth factor [HGF]/c-MET), suppresses 5′-AMP activated protein kinase, and alters Akt-mammalian target of rapamycin (mTOR) signaling cascades (17–21). Furthermore, the upregulation and recruitment of the membrane protein aquaporin 3 (AQP3) and the immunity protein mucin-13 (MUC13) have been observed (9, 10).

The recruitment of host organelles and related proteins to the PVM has also been reported during LS infection. The host Golgi and ER, as well as the vesicle markers ARF4, GBF1, GGA1, and COPI, accumulate around the PVM during development (22–25). Late endosomes and lysosomes (LAMP1/LAMP2), which carry proteins destined for degradation, also accumulate around the parasite. Although the exact mechanisms for vesicle cargo transfer to the parasite remain unknown, evidence suggests they can supply nutrients, including cholesterol (22, 26–29). Similarly, other host proteins (SCARB1, AQP3, APOH1, APPL1, and CERT1) may also help secure vital resources, but to date, only CERT1 has been linked to parasite acquisition of a specific nutrient (ceramide) (10, 30–33). In addition to these proteins, selective autophagy markers have been found at the PVM (LC3, p62, ubiquitin, NDP52, and NBR1), and are thought to contribute to nutrient acquisition and immune evasion (28, 34–37). Most recently, the SNARE proteins vesicle-associated membrane protein 7 (VAMP7), VAMP8, Vti1B, and Stx7 were shown to be recruited to the PVM to mediate lysosomal fusion (38). The growing, yet still small, list of host proteins associated with the PVM suggests that much remains to be discovered, including their recruitment mechanisms.

In this study, we sought to better understand the mechanism of host protein recruitment during the *Plasmodium* LS. To expand the current catalog of host proteins dysregulated during *P. berghei* infection, a comprehensive transcriptomic analysis was reanalyzed with a recent reference genome. This analysis and subsequent validation revealed that the host *CGA* gene is highly upregulated in *P. berghei*-infected cells across four host liver cell types, where protein recruitment to the PVM is observed in the late LS. CGA protein recruitment to the parasite and PV size was then quantified after depletion of 22 representative host membrane trafficking members using siRNAs. This study highlights the role of VAMP3 in CGA recruitment and *P. berghei* development. *VAMP3* depletion reduced PV size and increased LC3 accumulation, which resulted in parasite elimination from host cells after invasion. Confocal microscopy demonstrated that VAMP3 associates with the *P. berghei* and *P. falciparum* PVM, and ultrastructure expansion microscopy (U-ExM) suggests vesicle fusion may be occurring. We further show that VAMP3-positive vesicles accumulate at the PVM using a small-molecule probe, N-ethylmaleimide (NEM), further supporting host vesicle fusion to the host–pathogen interface. This work demonstrates the importance of the host secretory pathway to the *Plasmodium* liver stage, where a host SNARE protein facilitates growth and protects from host-mediated clearance.

## RESULTS

### RNA-seq identifies CGA as highly upregulated during *P. berghei* infection

**Fig 1:**
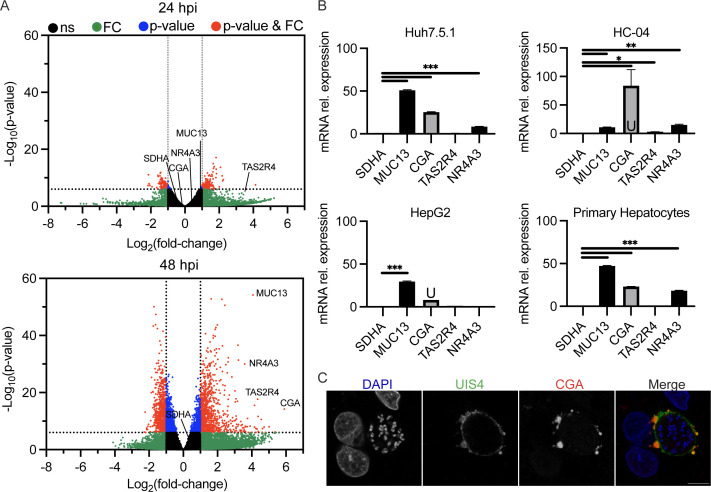
CGA is highly upregulated during liver stage *P. berghei* infection. (**A**) Volcano plot of differentially expressed genes at 24 and 48 hpi analyzing data from LaMonte et al. (9). Fold change infected vs uninfected is reported on the *X* axis, and the −log_10_ adjusted *P*-value on the *Y* axis. Dashed lines on the *X* axis indicate a cutoff for log_2_ fold change of >|1|, and a dashed line on the *Y* axis indicates a *P*-value cutoff of <1 × 10^−6^. Green dots indicate genes above fold change cutoff, blue dots show genes above the *P*-value cutoff, and red dots are genes above fold change and *P*-value cutoff values. (**B**) RT-qPCR of *MUC13, CGA, TAS2R4,* and *NR4A3* at 48 hpi in the indicated cell lines. *SDHA* was used as a control. Relative quantitation of the indicated host transcripts calculated using the ΔΔCT method (data presented as mean ± SEM, *n =* 3 biological replicates). Binding of CGA primers was observed only in infected samples from HepG2 and HC-04 cells, indicating high upregulation (U), but the mRNA relative expression is estimated based on the limit of detection. *P*-values display unpaired two-tailed t-test. *P < 0.05; **P < 0.01; ***P < 0.001. (**C**) Representative confocal microscopy images of HepG2 cells infected with *P. berghei* and stained with anti-PbUIS4 to visualize the PVM (green), anti-HsCGA (red), and DAPI (blue) at 48 hpi. Scale bar is 10 μm.

A reanalysis of a previous dual (parasite and human) RNA-seq study (9) with the rodent malaria parasite, *P. berghei* (*P. berghei*-GFP), identified a series of human transcripts that were upregulated or downregulated during LS infection at 24 and 48 hpi (Fig. 1A; Fig. S1A; Table S1). Different hepatoma host cells (HepG2s, Huh7.5.1s, and HC-04s) were used in these experiments, in which human cells were individually sorted into infected and uninfected pools based on the presence of a parasite-encoded GFP signal (Fig. S1B). *MUC13*, *CGA, TAS2R4*, and *NR4A3* were among the most upregulated, statistically significant protein-coding genes across all cell lines at 48 h post-infection (hpi).

To confirm upregulation of *MUC13, CGA, TAS2R4,* and *NR4A3* in *P. berghei*-infected primary human hepatocytes, Huh7.5.1, HepG2, and HC-04 cells, we completed a qRT-PCR validation study. Cells were infected with *P. berghei*-GFP parasites, sorted using flow cytometry at 48 hpi, and RNA was extracted from both infected and uninfected cells. From this, we observed consistent upregulation of *CGA,* which encodes a glycoprotein hormone, across all four studied cell lines (Fig. 1B). *CGA* and *NR4A3* were also reported to be upregulated in a 2022 scRNA-seq study on *P. berghei*-infected mouse liver at 36 hpi (Fig. S1C) (11). In this study, *MUC13* and *TAS2R4* were not detected and thus cannot be compared to our results. For subsequent studies, we prioritized *CGA* due to its robust upregulation across all four lines and the independent observation of its upregulation in mouse livers after *Plasmodium* infection.

We next sought to probe whether CGA has a functional role during *Plasmodium* infection. To accomplish this, HepG2 cells were treated with siRNA targeting *CGA* (siCGA) 48 h before infection, infected with luciferase-expressing *P. berghei* (*P. berghei*-Luc), and then host viability and parasite load were assessed at 48 hpi (Fig. S1D). A non-targeting scramble control (siCTRL) was included as a negative control, as well as an siRNA for a known host factor critical to *Plasmodium* LS invasion and development as a positive control (siSCARB1) (30). qRT-PCR demonstrated efficient mRNA knockdown of the targets (<5% expression), but *CGA* depletion did not impact HepG2 viability or *P. berghei* load, as assessed with the luciferase reporter, indicating that *CGA* is dispensable during infection. This finding is consistent with past RNA interference and CRISPR screens completed to discover host factors critical to LS *Plasmodium* (25, 39).

CGA protein localization during infection was visualized to further probe its potential function. HepG2 cells were infected with *P. berghei*-Luc, fixed at 48 hpi, and stained with anti-HsCGA and anti-PbUIS4, a known PVM resident protein (28). We observed accumulation of CGA in UIS4 labeled regions (PVM and membrane clusters), indicating that the host protein is recruited to the host–pathogen interface (Fig. 1C). No anti-HsCGA signal was observed in uninfected HepG2 cells, consistent with the RNA-seq results, or *P. berghei*-Luc-infected HepG2 cells that were treated with siCGA and fixed at 48 hpi, demonstrating antibody specificity (Fig. S1E).

### CGA recruitment to *Plasmodium* is affected by depletion of host vesicular network

**Fig 2:**
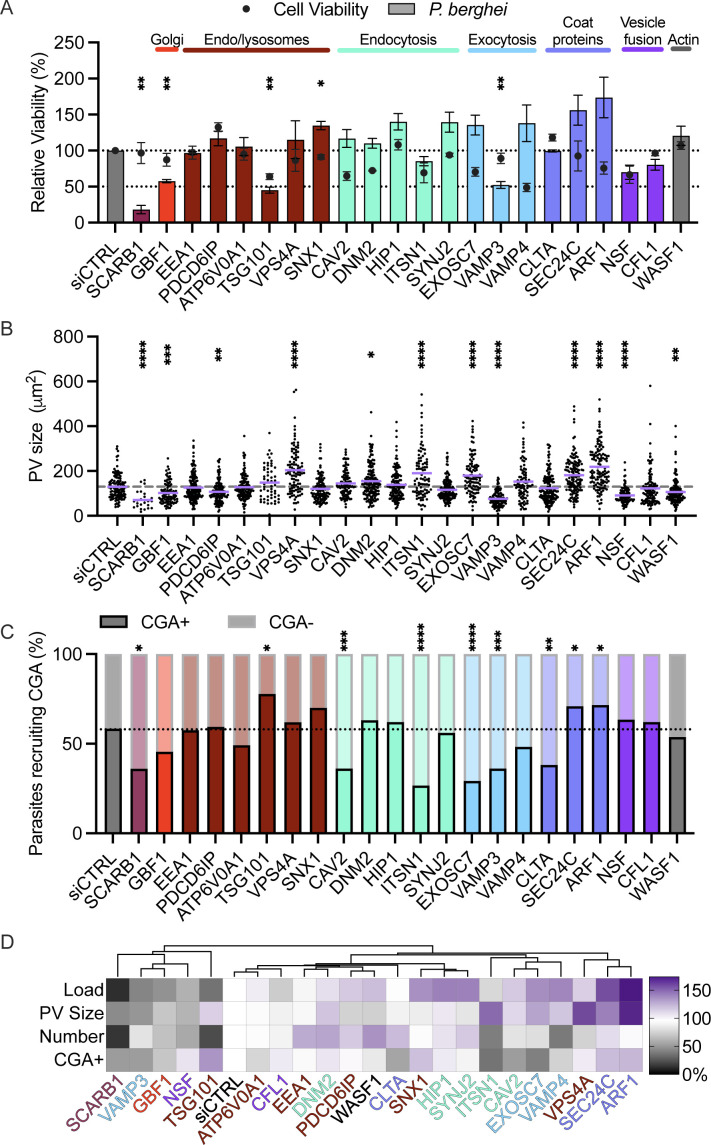
Host trafficking proteins are important for *P. berghei* LS development and CGA recruitment. (**A–C**) HepG2 cells were reverse-transfected with siRNAs targeting 22 human trafficking members involved in the Golgi (light red), endosomes/lysosomes (dark red), endocytosis (green), exocytosis (blue), coat proteins (light purple), vesicle fusion (dark purple), and actin (gray). Negative (siCTRL) and positive (SCARB1) controls were included. After 48 h, cells were infected with *P. berghei*-Luc. (**A**) HepG2 viability (black circles) and parasite load (bar) were measured at 48 hpi. Data were normalized to the negative control and shown as mean ± SEM. *n =* 3 biological replicates. *P*-values display Welch’s unpaired two-tailed t-test compared to siCTRL for *P. berghei* load. (**B**) Cells were fixed at 48 hpi, and the PVM was stained with anti-PbUIS4. PV size was quantified by measuring area within the PVM (FIJI). siCTRL (gray dashed line) and sample means (purple line) are indicated. *P*-values display two-way ANOVA compared to siCTRL. (**C**) Cells were fixed at 48 hpi and stained with anti-PbUIS4 and anti-HsCGA. Parasites were scored as positive or negative for CGA recruitment after visualization with confocal microscopy. *P*-values display the Cochran-Armitage test compared to siCTRL. (**B and C**) *n =* 2 biological replicates analyzing >80 PVs except SCARB1 *n =* 25 and TSG101 *n =* 54. (**D**) Euclidean cluster analysis using Morpheus (Broad Institute) online software highlights the importance of VAMP3, GBF1, and NSF. (**A–C**) *P < 0.05; **P < 0.01; ***P < 0.005; ****P < 0.001.

Given the robust gene upregulation and protein recruitment of CGA during *Plasmodium* LS infection, we sought to use it as a tool to investigate a possible connection between host protein recruitment to the PVM and vesicle-trafficking members. We investigated a panel of 22 representative genes involved in host vesicular trafficking, broadly surveying the Golgi, endosomes/lysosomes, endocytosis, exocytosis, coat proteins, vesicle fusion, and actin. Genes were prioritized for selection if they were previously implicated in *Plasmodium* or other host–pathogen systems as being important for infection (Table S2). As a first step, the impact of siRNA knockdown of the 22 selected genes on host viability and parasite load was measured. HepG2 cells were reverse-transfected with two pooled siRNAs targeting host trafficking genes at 25 nM for 48 h and then infected with *P. berghei*-Luc (Fig. 2A). The negative scrambled control (siCTRL) and positive control (siSCARB1) were also included, and hepatocyte viability and parasite load were assessed at 48 hpi. At the time of infection, identically treated cells on a separate plate were harvested, and the relative mRNA expression for each gene was assessed using qRT-PCR. The expression of each studied gene was significantly reduced relative to the scrambled siRNA negative control (<30% expression) (Fig. S2A). Host toxicity was observed with depletion of some genes, which limited our ability to assess their impact on parasite load. For example, *TSG101* (endosome/lysosome) depletion reduced parasite load and host cell viability. In contrast, *GBF1* (Golgi pathway) and *VAMP3* (exocytosis pathway) depletion reduced parasite load without significantly impacting HepG2 viability, while *SNX1* (endosome/lysosome) reduction increased parasite load (Fig. 2A).

To assess the impact on PV size and number (PVs/well) after depletion of host vesicular trafficking members, a phenotypic analysis was conducted. In 384-well glass-bottom plates, reverse transfections were performed in a pooled format, and cells were infected with *P. berghei*-Luc. After 48 hpi, cells were fixed and stained with DAPI to visualize nuclei and anti-PbUIS4 to measure the PV size (Fig. 2B). We observed that *GBF1* and *SCARB1* depletion decreased PV size, consistent with previously published studies (25, 30). Additionally, depletion of *PDCD6IP* (*ALIX*), *VAMP3*, *NSF*, and *WASF1* significantly decreased PV sizes at 48 hpi. Remarkably, we also identified several conditions (*VPS4A*, *DNM2*, *ITSN1*, *EXOSC7*, *SEC24C*, and *ARF1* reduction) that increased PV size. The number of PVs in each well was also determined, which identified conditions that decreased parasite numbers (*TSG101*, *ITSN1*, *VAMP4,* and *NSF* reduction), similar to the positive control *SCARB1*, and that increased parasite numbers (*EEA1*, *CLTA,* and *WASF1* reduction) compared to the negative control (Fig. S2B). As this assessment is at 48 hpi, changes in parasite invasion and/or immune-mediated clearance could influence these numbers.

Lastly, host protein recruitment to *P. berghei* after knockdown of the 22 panel members was investigated. To accomplish this, we used the aforementioned *P. berghei*-infected HepG2 cells in 384-well glass-bottom plates and stained with anti-HsCGA, thereby using it as an indicator for broad perturbation of host protein recruitment (Fig. S2C). As a binary assessment, each parasite was scored by observation as positive or negative for CGA colocalization with UIS4, where a low variability between technical and biological replicates was observed (Fig. 2C; Fig. S2D). In siCTRL cells, CGA is recruited to ~60% of the PVs at 48 hpi, and most of the conditions tested did not impact this recruitment. However, recruitment was statistically decreased with *SCARB1*, *CAV2*, *ITSN1*, *EXOSC7*, *VAMP3*, and *CLTA* reduction. Interestingly, four out of the six noted genes (*CAV2*, *ITSN1*, *EXOSC7*, and *VAMP3*) are involved in endocytosis or exocytosis processes. Additionally, an increase in CGA recruitment was observed with *TSG101*, *SEC24C*, and *ARF1* reduction, implicating coat proteins in this process. To understand whether CGA recruitment directly correlates with PV size, we compared the PV size of parasites observed with and without CGA in the siCTRL sample (Fig. S2E). CGA recruitment did not correlate with PV size, further indicating a dispensable role for this protein.

To synthesize all the data acquired, we represented percent parasite load, PV size, parasite number (PVs/well), and CGA recruitment as a heatmap (Fig. 2D). Genes were clustered based on shared phenotypes, according to Euclidean distance (40). Overall, this highlighted distinct phenotypes induced by disruption of host vesicular pathways, where only *SCARB1* (positive control), *VAMP3,* and *GBF1* depletion decreased all four parameters studied. In contrast, *SEC24C* and *ARF1* reduction increased PV size, parasite numbers, and CGA recruitment, suggesting coat proteins play a role during LS infection. As GBF1 has been previously investigated by our lab, we were particularly intrigued by the role of VAMP3 in LS *Plasmodium* (24, 25). Thus, two individual siRNAs targeting *VAMP3* were then tested for their impact on parasite load. Both siRNAs reduced *VAMP3* levels and reduced *P. berghei* parasite load in HepG2 cells without cytotoxicity (Fig. S3A). As a result, VAMP3 was selected for further investigation.

### VAMP3 is important for *Plasmodium* development

**Fig 3:**
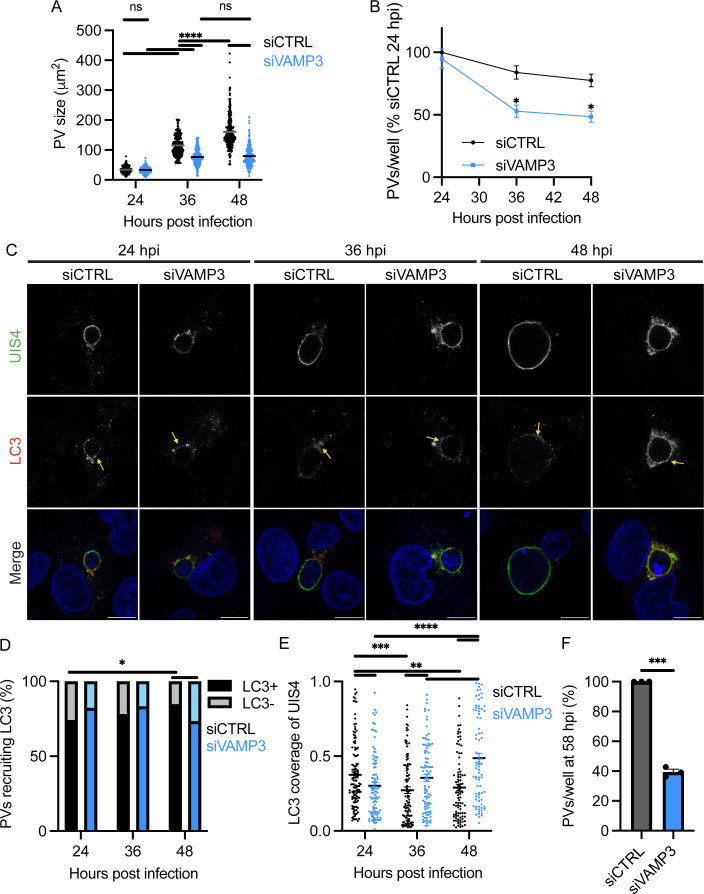
*VAMP3* depletion causes growth defects and increases LC3 recruitment that leads to *Plasmodium* clearance. (**A–E**) HepG2 cells were reverse-transfected with siCTRL or siVAMP3 (25 nM) for 48 h before infection with *P. berghei*-Luc. Cells were fixed at 24, 36, and 48 hpi and stained for anti-PbUIS4, anti-HsLC3, and DAPI. (**A**) PV size determined by measuring area within PVM via anti-UIS4 (Aivia 14 software). *n =* 3 biological replicates analyzing >200 PVs. *P*-values display Welch’s unpaired two-tailed t-test. (**B**) The number of PVs per well was counted and normalized to the 24 hpi siCTRL wells. *n =* 3 biological replicates analyzing 3–6 wells. *P*-values display Welch’s unpaired two-tailed *t*-test. (**C**) Representative confocal images displaying PbUIS4 (green), HsLC3 (red), and DAPI (blue). Yellow arrows indicate areas of LC3 and UIS4 colocalization. Scale bar is 10 μm. (**D**) LC3 recruitment where PVs positive for LC3 had >1 µm^2^ overlap in signal between UIS4 and LC3 (Aivia 14 software). *n =* 3 biological replicates analyzing >100 PVs. *P*-values display the Cochran-Armitage test. (**E**) Overlap coefficient (Aivia 14 software) of HsLC3 and PbUIS4 was plotted for PVs that were positive in D at 24, 36, and 48 hpi. *P*-values display unpaired two-tailed Mann-Whitney. (**F**) HepG2 cells were reverse-transfected with siCTRL or siVAMP3 for 48 h, infected with *P. berghei*-Luc, and fixed at 58 hpi. Parasites were stained with PbUIS4, and the number of PVs per well was determined to assess clearance by normalizing each replicate to the siCTRL wells. *n =* 3 biological replicates analyzing 3–6 wells. *P*-values display Welch’s unpaired two-tailed *t*-test. (**A–F**) ns = non-significant; *P < 0.05; **P < 0.01; ***P < 0.005; ****P < 0.001.

To better understand the relationship between VAMP3 function and parasite development, we performed a temporal PV size study in HepG2 cells treated with siVAMP3 and a scrambled control before *P. berghei*-Luc infection. After siRNA transfection and *P. berghei*-Luc infection, cells were fixed at 24, 36, and 48 hpi and stained with DAPI and anti-PbUIS4. *VAMP3* knockdown was confirmed with qRT-PCR, and the PV size was measured at each timepoint (Fig. 3A). At 24 hpi, at the start of replication when the parasite is small and contains a small number of nuclei, the PV size was not impacted by *VAMP3* depletion (*P* = 0.098). PV size then increased in both conditions between 24 and 36 hpi, when the parasites replicate and expand; however, the magnitude of this growth was less in *VAMP3*-depleted cells when compared to the control. Furthermore, growth in *VAMP3*-depleted cells was arrested between 36 and 48 hpi (*P* = 0.281), which contrasts with the significant, and expected, increase in PV size observed in control cells. Thus, while a growth restriction is observed between 24 and 36 hpi, the largest arrest occurs in *VAMP3*-deficient cells between 36 and 48 hpi, when no growth is observed.

To further understand the effects of *VAMP3* depletion on LS *P. berghei*, the number of parasites per well was counted at 24, 36, and 48 hpi (Fig. 3B; Fig. S3B through D). At 24 hpi, there was no significant difference between siCTRL and siVAMP3 wells (*P* = 0.573), but there was a statistically significant decrease at 36 (*P* < 0.05) and 48 hpi (*P* < 0.05) to about 50% of the negative control at 24 hpi. With this more rigorous follow-up assay (three biological replicates with six technical wells), we were able to detect a statistical decrease upon *VAMP3* depletion, which was previously obscured (Fig. S2B) (two biological replicates with two technical wells). While our analysis could be influenced by host or parasite effects with *VAMP3* depletion, our cytotoxicity assays indicate that gene depletion did not impact HepG2 viability, a finding consistent with our confluency assessment for each well. This supports a direct or indirect role for VAMP3 in parasite survival, which could be by supplying nutrients, as parasites that fail to develop are eliminated, and/or by supporting evasion of the host immune response.

### *VAMP3* depletion increases LC3 recruitment that leads to *Plasmodium* clearance

*VAMP3* depletion has been shown to alter many aspects of mammalian cells, including membrane receptor trafficking and autophagosome formation (41–43). Additionally, past studies have shown a direct correlation between *Plasmodium* LS development and the autophagosome marker, microtubule-associated protein light chain 3 (LC3) (37, 44, 45). Specifically, LC3 sequestration by the parasite is necessary to avoid elimination.

To test a possible link between LC3 and VAMP3, HepG2 cells were treated with siVAMP3 or a scrambled negative control before infection with *P. berghei*-Luc*,* and then stained with DAPI, anti-PbUIS4, and anti-HsLC3 antibodies at 24, 36, and 48 hpi. *VAMP3* knockdown was confirmed with qRT-PCR, and protein recruitment was assessed with confocal microscopy. Consistent with previous reports, we observed LC3 accumulation around the PVM at each timepoint (Fig. 3C). As expected, LC3 staining was not observed in uninfected HepG2 cells (Fig. S3E). We then used Aivia software to determine how many PVs recruit LC3 after siCTRL and siVAMP3 treatment. After thresholding each channel, PVs with >1 µm^2^ LC3 signal overlapping with UIS4 signal were considered LC3+. At 24 and 36 hpi, no significant difference in LC3 recruitment was observed between treatments, but a greater number of PVs recruited LC3 in siCTRL cells (84%) than in siVAMP3 cells (74%) at 48 hpi (Fig. 3D).

To gain a deeper understanding of LC3 recruitment, we performed an LC3/UIS4 overlap analysis, quantifying the amount of the PVM covered by LC3 at each timepoint (Fig. 3E). At 24 hpi, the LC3/UIS4 overlap on the PVM in siVAMP3 cells is less than the overlap on the PVM in siCTRL cells. At 36 hpi, this phenotype shifts such that PVMs in control cells have a lower coverage with LC3 than in siVAMP3 cells. This trend continues and becomes more apparent at 48 hpi, where PVMs in *VAMP3*-depleted cells have higher LC3 coverage than in the control cells. Past reports have shown that healthy parasites have PVMs coated with LC3 at 6–40 hpi, but they shed the protein 40–48 hpi to avoid elimination (34, 35). In contrast, we observe an increase in LC3 coverage with *VAMP3* depletion at later timepoints, suggesting parasites in these cells may be less efficient at avoiding clearance by LC3 shedding. To test this, cells were treated with siCTRL or siVAMP3, infected with *P. berghei*-Luc*,* and stained with DAPI and anti-PbUIS4 at 58 hpi to determine parasite numbers (PVs/well) by microscopy. This late-stage LS development was selected as it allows for greater clearance to occur when compared to the previously assessed 48 hpi timepoint. From this, we observed significantly fewer PVs in *VAMP3*-depleted cells when compared to the negative control, supporting the hypothesis that *VAMP3* depletion increases susceptibility to host-mediated clearance (Fig. 3F; Fig. S3F).

**Fig 4:**
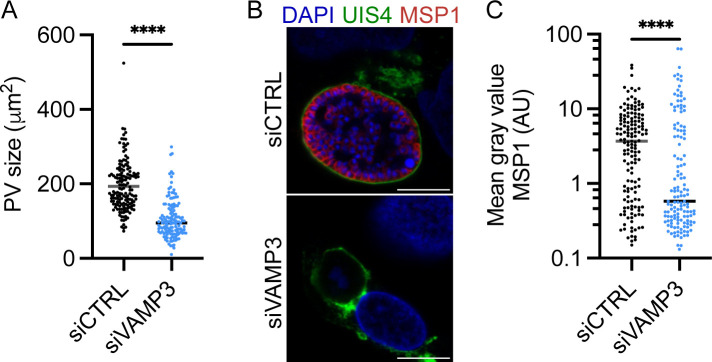
Parasites in *VAMP3*-depleted cells are developmentally impaired. (**A–C**) HepG2 cells were reverse-transfected with siCTRL or siVAMP3 for 48 h, infected with *P. berghei*-Luc, and fixed at 58 hpi. (**A**) Parasites were stained with anti-PbUIS4, and the PV size was determined (Aivia 14 software). *P*-values display Welch’s unpaired two-tailed *t*-test. (**B**) Representative confocal microscopy images of parasites stained with anti-PbUIS4 (green), anti-PbMSP1 (red), and DAPI (blue). Scale bar is 10 μm. (**C**) Mean gray value (AU) of the MSP1 channel within the PVM was quantified (Aivia 14 software). *P*-values display unpaired two-tailed Mann-Whitney test. (**A and C**) *n =* 3 biological replicates analyzing >130 PVs. ****P < 0.001.

In addition to replication, parasites mature into blood-infective merozoites during the late liver stages. This maturation is characterized by expression of merozoite surface protein 1 (MSP1) on the surface of merozoites as they prepare for egress. To assess whether LS parasites are properly developing in *VAMP3*-depleted cells, PV size was quantified, and MSP1 expression was assessed at 58 hpi. Similar to our observations at 48 hpi, PV size decreased with *VAMP3* depletion at 58 hpi (Fig. 4A). At this time, most parasites in siCTRL cells displayed MSP1 staining within the PV around individual nuclei as expected, while staining was reduced with *VAMP3* depletion (Fig. 4B; Fig. S4). Quantification of the mean gray value of the MSP1 signal within the PV demonstrated that parasites in *VAMP3*-depleted cells exhibit significantly lower MSP1 levels (Fig. 4C). These observations suggest that parasite development during the LS is impaired without host VAMP3.

### *Plasmodium* recruits VAMP3 during LS infection

**Fig 5:**
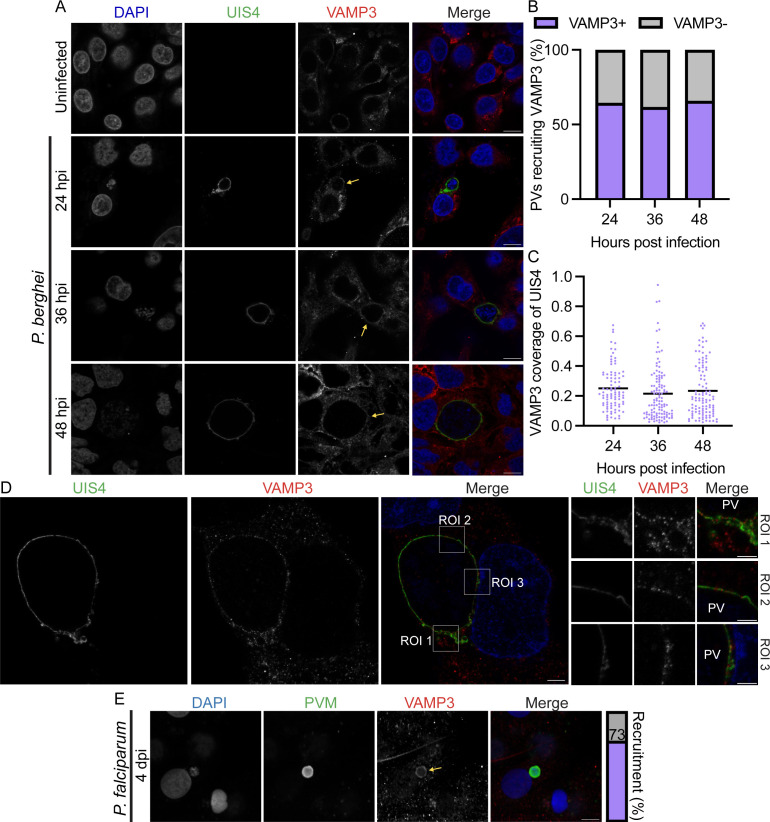
VAMP3 is recruited to *P. berghei* and *P. falciparum*. (**A**) Representative confocal microscopy images of uninfected and *P. berghei-*infected HepG2 cells fixed at 24, 36, or 48 hpi. Cells were stained with anti-PbUIS4 (green) to visualize the PVM, anti-HsVAMP3 (red), and DAPI (blue). Yellow arrows indicate areas of VAMP3 and UIS4 colocalization. Scale bar is 10 μm. (**B**) VAMP3 recruitment was assessed for PVs where >1 µm^2^ overlap in UIS4 and VAMP3 signal (Aivia 14 software) was assigned positive at 24, 36, and 48 hpi. (**C**) Overlap coefficient (Aivia 14 software) was plotted for individual PVs that were positive in B at 24, 36, and 48 hpi. (**B and C**) *n =* 3 biological replicates analyzing > 140 PVs. (**D**) Representative confocal microscopy images of *P. berghei*-infected HepG2 cells fixed at 48 hpi and expanded 4.5-fold using an acrylamide hydrogel. Cells were stained with anti-PbUIS4 (green) to visualize the PVM, anti-VAMP3 (red), and DAPI (blue). Scale bar is 10 μm and has not been adjusted for expansion. Insets show 3 regions of interest (ROI) with a scale bar of 5 μm, also not adjusted for expansion. (**E**) Representative confocal image of *P. falciparum-*infected PHHs at 4 dpi stained with anti-PbUIS4 (green) to visualize the PVM and HsVAMP3 (red). VAMP3 and UIS4 colocalization is observed in 73% of parasites. *n* > 50 PVs. Yellow arrow indicates an area of VAMP3 and PVM colocalization. Scale bar is 10 μm.

We investigated VAMP3 localization throughout the *P. berghei* LS. HepG2 cells were infected with *P. berghei*-Luc and stained with DAPI, anti-PbUIS4, and anti-HsVAMP3 antibodies at 24, 36, and 48 hpi for confocal microscopy. In uninfected HepG2 cells, VAMP3 was observed in the host Golgi, as expected, but staining was also observed around the PVM in *P. berghei*-infected cells at each timepoint (Fig. 5A; Fig. S5). No antibody signal was observed in HepG2 cells treated with siVAMP3, demonstrating the anti-HsVAMP3 antibody specificity (Fig. S6). To quantify the prevalence of VAMP3 recruitment to the PVM, parasites were scored as VAMP3+ or VAMP3− based on overlap with UIS4. We observed that VAMP3 is robustly recruited (>50%) at all three timepoints (Fig. 5B). To evaluate the degree of recruitment for a given parasite, the percent coverage of VAMP3 on UIS4 was determined. Here, we observed consistent colocalization at each timepoint at around 20% of the PVM (Fig. 5C).

We questioned whether VAMP3 fuses with the PVM, or if distinct VAMP3 vesicles are in proximity to the membrane without fusion. To address this question, ultrastructure expansion microscopy (U-ExM) was employed to obtain super-resolution quality images of VAMP3 during infection. Using a pre-gelation staining protocol previously reported by Atchou et al., *P. berghei*-infected HepG2 cells were physically expanded ~4.5-fold before visualization with confocal microscopy (46). Cells were fixed at 48 hpi and stained with DAPI, anti-PbUIS4, and anti-HsVAMP3 before gelation. With this expansion, we observed distinct vesicles that were recruited to the PVM. Furthermore, a smooth VAMP3 signal that colocalized with UIS4 was also observed, suggesting vesicle integration had occurred (Fig. 5D; Fig. S7).

To determine whether VAMP3 recruitment is conserved in the human-infective *P. falciparum*, localization was assessed in infected primary human hepatocytes 4 days post-infection (dpi). This timepoint represents early schizogony, which is comparable to ~24–30 hpi in *P. berghei* parasites. Although *P. falciparum* does not encode UIS4, the PbUIS4 antibody can cross-react with an unknown protein at the *P. falciparum* PVM and can be used to mark the membrane. We observed robust VAMP3 recruitment (73% of PVs were VAMP3+) in *P. falciparum*-infected PHHs, consistent with our observations in *P. berghei*-infected HepG2 cells (Fig. 5D; Fig. S8).

We next sought to assess whether *P. berghei* viability is required for VAMP3 recruitment. To test this, parasites were treated with atovaquone, which disrupts the mitochondrial electron transport chain, or cabamiquine, which halts parasite protein translation, from 24 to 48 hpi, and compared to a negative DMSO control. Measurement of the PV size at 48 hpi was assessed first, which validated that atovaquone and cabamiquine were restricting PV growth as expected (Fig. S9A). LC3 recruitment with compound treatment was next assessed as a control since the protein is actively shed by the parasites between 24 and 48 hpi. We observed that DMSO-treated parasites shed LC3 between 24 and 48 hpi, while atovaquone- and cabamiquine-treated parasites maintained LC3 coverage (Fig. S9B and C). VAMP3 recruitment after *P. berghei* treatment with atovaquone or cabamiquine was then investigated, where we observed it was reduced at 48 hpi when compared to the DMSO control. These observations suggest that VAMP3 recruitment is dependent on *P. berghei* viability at 24–48 hpi (Fig. S10).

### VAMP3 vesicles accumulate at the PVM with NEM treatment

To probe whether VAMP3 vesicles fuse to the PVM, a chemical biology strategy was employed. Prior research has demonstrated that the small molecule NEM disrupts SNARE-mediated processes by irreversibly binding to N-ethylmaleimide sensitive factor (NSF), which plays a critical role in vesicle recycling (47). Specifically, NEM allows SNARE vesicles to bind and fuse to target membranes but inhibits vesicle fission, resulting in VAMP3/SNARE accumulation on target membranes (Fig. S11A). Thus, if VAMP3 vesicles fuse to the PVM, an increase in overlap of VAMP3 with UIS4 would be observed after NEM treatment. NEM is highly reactive and will bind to other cysteine residues during the incubation window; however, our microscopy-based assay only monitors VAMP3 localization and therefore cannot readily detect other cellular changes.

**Fig 6:**
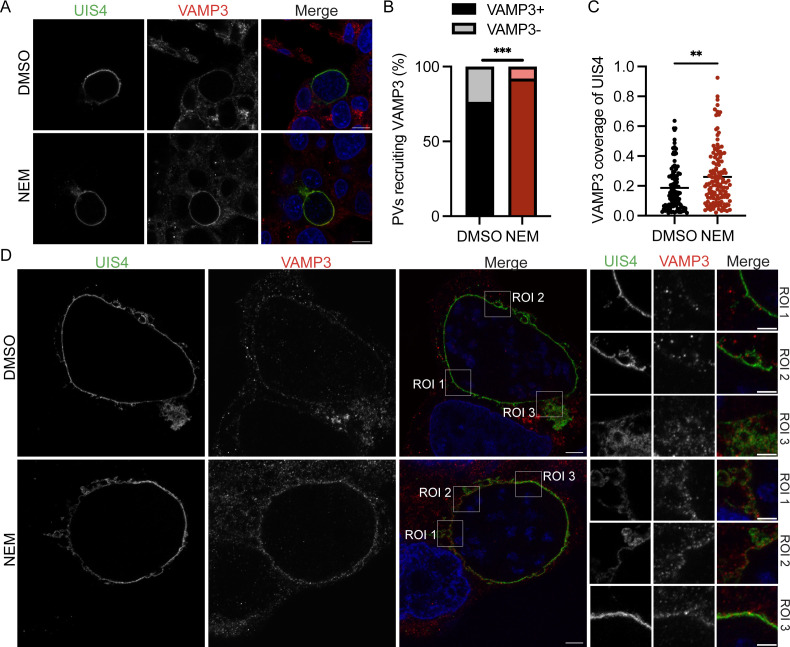
VAMP3 vesicles accumulate at the PVM with NEM treatment. (**A**) Representative confocal microscopy images of *P. berghei*-infected HepG2 cells treated with DMSO or 250 μM NEM from 47.5 to 48 hpi and fixed at 48 hpi. Cells were stained with anti-PbUIS4 (green), anti-HsVAMP3 (red), and DAPI (blue). Scale bar is 10 μm. (**B**) VAMP3 recruitment was assessed for PVs where >1 µm^2^ overlap in UIS4 and VAMP3 signal (Aivia 14 software) was assigned positive. *P*-values display the Cochran-Armitage test. (**C**) Overlap coefficient (Aivia 14 software) was plotted for individual PVs that were positive in panel B. *P*-values display unpaired two-tailed Mann-Whitney test. (**B and C**) *n =* 3 biological replicates analyzing >130 PVs. (**D**) Representative confocal microscopy images of *P. berghei*-infected HepG2 cells treated with DMSO or 250 μM NEM from 47.5 to 48 hpi and fixed at 48 hpi. Cells were stained with anti-PbUIS4 (green) to visualize the PVM, anti-VAMP3 (red), and DAPI (blue) and expanded 4.5-fold using an acrylamide hydrogel. The scale bar is 10 μm and has not been adjusted for expansion. Insets show 3 ROIs with a scale bar of 5 μm, also not adjusted for expansion. **P < 0.01; ***P < 0.005.

*P. berghei*-infected HepG2 cells were treated with the negative DMSO control or 250 μM NEM from 47.5 to 48 hpi in 384-well plates. At 48 hpi, cells were fixed, stained with DAPI, anti-PbUIS4, and anti-HsVAMP3, and then imaged with confocal microscopy (Fig. 6A; Fig. S11B). In uninfected cells, an increase in VAMP3 accumulation at the Golgi was observed, consistent with previous reports (Fig. S11B). The entire well was also imaged to assess cell confluency, where no difference was observed between DMSO- and NEM-treated cells (Fig. S12). These optimized treatment conditions are consistent with past studies using NEM to probe vesicle fusion events (47). Of note, we observed that higher NEM concentrations or longer incubation times resulted in reduced cell confluency and/or disruption of PVM integrity and thus could not be used. Every PV in the well was then scored as VAMP3+ or VAMP3−, where we observed a significant increase in VAMP3+ PVs with NEM treatment compared to the DMSO control (*P* < 0.005) (Fig. 6B). To evaluate the degree of VAMP3 recruitment for each PV, the overlap coefficient of VAMP3 on UIS4 was determined. Here, an increase in VAMP3 coverage on the PVM was observed with NEM treatment compared to the DMSO control (Fig. 6C). Pre-gelation expansion microscopy was next used to investigate VAMP3 at the PV after NEM treatment at higher resolution (Fig. 6D; Fig. S13). We observed greater accumulation of VAMP3, both punctate vesicles and smooth signal, near the PVM with NEM treatment compared to the DMSO control. Both the increase in parasites recruiting VAMP3 and the increase in the amount of VAMP3 on the PVM after NEM treatment support the proposal that VAMP3 vesicles fuse to the PVM.

## DISCUSSION

*Plasmodium* is an intracellular pathogen that relies on its host cell for development and replication. Though it is known that the parasite induces the expression and recruitment of host factors, the mechanistic details surrounding these events remain elusive. In this study, we show that liver stage *Plasmodium* parasites rely on host SNARE vesicles to support development and survival.

Previous RNA-seq studies have demonstrated the broad reprogramming of host genes throughout *Plasmodium* LS infection (8, 9). Our analysis further identifies genes upregulated and downregulated at 24 and 48 hpi in hepatoma cells infected with *P. berghei*. Beyond a single cell line, we profiled their expression across various hepatoma cells as well as primary hepatoma cells infected with *P. berghei* by RT-qPCR. The most consistently upregulated gene, *CGA*, encodes the common alpha chain of at least four glycoprotein hormones and, when combined with various beta chains, plays an important role in host cell endocrine signaling. Surprisingly, CGA did not appear to be essential for parasite infection. Despite this, it would be valuable to investigate other genes identified in our analysis, especially those whose functions are clearly linked to key aspects of parasite infection. For example, the eukaryotic transcription factor *NR4A3* was upregulated in multiple hepatocyte-derived lines. Although its exact function in mammalian cells is unknown, previous reports suggest that it can control glucose levels, a valuable nutrient for *Plasmodium* (48). In addition, several noncoding genes were in the set of significantly upregulated genes, including the long-noncoding RNAs (lncRNAs) *NEAT1* and *MALAT1* (also known as *NEAT2*) (Fig. S14). Both of these lncRNAs have been implicated in inflammasome activation, presenting a compelling hypothesis that they may be associated with host-mediated clearance (49–51). This highlights the broad reprogramming of liver cells that *Plasmodium* must perform for its survival. Future studies could focus on these factors and resolve their function during infection.

Although host proteins are known to associate with the PVM during the LS, the mechanisms of this recruitment remain unclear. Past studies suggest nutrients (proteins, lipids, and small solutes) are delivered to the parasite through autophagy, due to the many autophagic proteins (i.e., LC3, NDP52, NBR1, ubiquitin, p62, SQSTM1, and LAMP1) that decorate the PVM (26, 28, 29, 34–37). One caveat to this theory for host protein recruitment is that proteins are denatured and degraded into their building blocks during autophagy; thus, functional proteins cannot be delivered through this route. Instead, we hypothesized that *Plasmodium* obtains intact host proteins via membrane trafficking sources, which is supported by studies demonstrating vesicle proteins (COPB2, GGA1, ARF4, GBF1, Stx7, Vti1B, VAMP7, and VAMP8) at the PVM (24, 25, 38, 39).

To investigate the role of host membrane trafficking in the *Plasmodium* LS, we used siRNAs to deplete a panel of host genes. Given that these experiments relied on siRNA-mediated depletion of trafficking proteins, the observed changes in PV size and parasite load could stem from direct or indirect effects of the altered host gene expression. To avoid genes that may impact *Plasmodium* by generally disrupting the host cell, we prioritized candidates that did not inhibit host cell viability after siRNA treatment. Our data highlighted the importance of host VAMP3 for parasite development and CGA recruitment. Beyond VAMP3, our study uncovered a cluster that included VPS4A and the coat proteins ARF1 and SEC24C, which increased PV size. The coat proteins in this cluster also enhanced *Plasmodium* CGA recruitment. *VPS4A* depletion can impair autophagic flux, leading to endosome accumulation within the cell; a process that has been linked to LS parasite development (52). This leads us to speculate that overcompensation mechanisms, which are well documented with the Arf family, are influencing our observations (53, 54). In support of this proposal, De Niz et al. previously showed that ARF1 was essential for parasite development and survival through the use of an overexpressed dominant negative (ARF1 T31N) (23). The dominant negative was perhaps less susceptible to an overcompensation mechanism since it was ectopically overexpressed and did not affect *ARF1* transcripts or protein levels within the cell. Thus, we believe our observed promotion of parasite growth stems from upregulation of compensatory pathway members after *ARF1* (and *SEC24C*) knockdown. This could be tested with RNA sequencing after gene knockdown or by completing experiments in a conditional knockdown cell line. The endosomal sorting complexes required for transport (ESCRT) proteins (TSG101, VPS4A, and PDCD6IP/ALIX) are also an interesting family for future study. In the related apicomplexan parasite, *Toxoplasma gondii*, host ESCRT machinery is used to gather cytosolic proteins as a nutrient source for *T. gondii* development (55, 56). Here, we observed that *PDCD6IP* depletion reduced PV size, but *TSG101* depletion had no change, and *VPS4A* depletion increased PV size. It would be interesting to establish if only some host ESCRT proteins are used by the parasite, or if compensatory mechanisms are confounding the results.

Our study focused on VAMP3, a protein responsible for many cellular functions including exocytosis (the Golgi to plasma membrane) and recycling (plasma membrane to recycling endosomes and the Golgi) pathways (57–59). In Fig. 2C, we show that trafficking proteins from the exocytosis pathway (two of three studied) decreased CGA recruitment. Thus, VAMP3 vesicles originating from the Golgi and traveling to the plasma membrane may be redirected to the parasite. Past studies showing the recruitment and integral role of GGA1 (Golgi to endosome) are consistent with this proposal (25). Also, other proteins that are secreted or localized to the plasma membrane, like MUC13 and AQP3, have been reported on the PVM (9, 10). In contrast, recycling endosomes (marked with the transferrin receptor) have not been shown to associate with the *P. berghei* PVM, suggesting that our observations on VAMP3 vesicles originating from the Golgi are not a general feature of all host membrane-bound vesicles. Future studies using live microscopy would be valuable to clarify the question of vesicle transport direction (26). Additionally, since VAMP3 localization to the PVM was influenced by parasite metabolic activity, the parasite likely plays an active role in recruiting VAMP3 vesicles to the PVM. In addition to this active recruitment, host-driven VAMP3 association may also be occurring, which would be interesting to probe in future experiments.

We speculate that VAMP3 vesicles could provide nutrients (either proteins, lipids, or other small molecules) to LS *Plasmodium*. This is consistent with VAMP3 recruitment during the mid and late LS, the observed growth defect with knockdown, and the hindered development of blood-infective merozoites. We also observed a decrease in PVs/well with *VAMP3* depletion, with no effect on invasion (based on the 24 hpi parasite number). This decrease could be parasite death due to a lack of nutrients, either directly or indirectly, if arrested parasites cannot evade the host cell immune response. We probed the latter possibility by co-staining with host LC3, whose interactions with the PVM are well studied (28, 34, 35, 37, 45, 60). LC3 is recruited to the PVM early in infection but is shed by healthy parasites after 40 hpi as a cellular immune evasion strategy; if shedding fails by 48 hpi, parasites are cleared (35). Our observations with *Plasmodium*-infected *VAMP3*-depleted cells are consistent with this process, where LC3 coverage increased throughout infection while parasite numbers decreased up to 58 hpi relative to the control. However, our study cannot determine whether unhealthy parasites were eliminated because of nutrient deprivation, an inability to shed LC3 and thereby evade clearance, or a potential interaction between these processes. Interestingly, we observed no difference in size between VAMP3+ and VAMP3− PVs. Given that our analysis was completed on fixed cells, transient interactions could have been missed, or VAMP3 could indirectly contribute to LS development. To address this, future live cell imaging experiments could be completed to directly assess the link between VAMP3 recruitment and PV size.

Completing *Plasmodium* LS studies in VAMP3 knockout (KO) cells or mice would also be valuable, but past studies have shown that stable KO of VAMP3 results in a loss of the knockdown phenotype (membrane receptor recycling), as the system likely adapts over time to use other VAMPs (VAMP2/7/8) (59, 61). Additionally, there are likely redundancies in SNARE protein functions. This was recently reported for VAMP7, VAMP8, and Vti1B during LS *P. berghei* infection, where single KOs had no effect on the parasite but double and triple KOs restricted PV size (38). In this report, the authors demonstrated the recruitment of these host proteins to the PVM; however, when GFP-VAMP3 was overexpressed in HeLa cells, VAMP3 recruitment to the PVM was not observed at 24 hpi. Our study utilized a monoclonal VAMP3 antibody to investigate endogenous VAMP3 localization in HepG2 cells; thus, the discrepancy in findings could be due to the different cell lines or detection methods used. Nonetheless, these studies collectively underscore the importance of the SNARE protein family to the *Plasmodium* LS.

The identification of cargo within VAMP3 vesicles remains a pressing question that is challenged by the dissection of live mosquitoes to conduct infections and the low *in vitro* infection rate of hepatocytes. Despite these challenges, it would be interesting to evaluate whether other proteins known to localize to the PVM, including MUC13 and AQP3, are likewise delivered by VAMP3+ vesicles. Additionally, proximity labeling studies like Selective Proteomic Proximity Labeling using Tyramide (SPPLAT) or Tyramide Signal Amplification Mass Spectrometry (TSA-MS) could offer an unbiased approach to identify the proteins within these vesicles (62, 63). Since CGA was dispensable for *P. berghei* infection and PV size, perhaps this technique could identify a protein contained within VAMP3 vesicles that is essential to the parasite.

*Plasmodium* parasites are auxotrophic and rely heavily on the host cell for nutrients during both the liver and blood stages. Past studies have proven uptake of BSA-gold particles into the PV space using transmission electron microscopy, but the mechanism remains elusive (26). Additionally, the authors’ electron micrographs showed close proximity of late endosomes and vesicles, but they could not find direct evidence of fusion events (26). As previously mentioned, the ESCRT pathway could provide nutrients from the host cytosol, but there are no studies investigating the potential role of ESCRT during *P. berghei* infection. An alternative route is vesicle/organelle fusion events, either incomplete, short-lived “kiss-and-run” fusion or full fusion. Interestingly, SNARE complexes control the degree of fusion events on membranes, where incomplete zippering of the vesicle and target membranes results in narrow and unstable pores (“kiss-and-run”), and complete zippering results in full fusion and vesicle collapse into the target membrane (64). One lab has experimentally proven that “kiss-and-run” fusion events are used to deliver cholesterol to the PVM from late endosomes (27). Here, we were able to show that VAMP3 accumulates near the PVM, which could indicate full zippering and vesicle collapse. Future experiments using live cell microscopy and fluorescence recovery after photobleaching (FRAP) would provide additional insights into this mechanism.

In the host SNARE family, we tested VAMP3, VAMP4, and NSF. Both *VAMP3* and *NSF* depletion decreased PV size, while no impact was observed with *VAMP4* depletion. Another SNARE protein, VAMP7, is recruited to the *P. yoelii* PVM at 48 hpi and is important for invasion, showing a potential dual role for the protein during infection (65). It is enticing to speculate that the *Plasmodium* LS expresses effector proteins or PVM-resident proteins to influence host vesicle recruitment and/or fusion. While the *P. falciparum* blood stages are known to export >400 proteins into the host red blood cell, only a small number of exported proteins (CSP, LISP2, and SLTRiP) have been reported in the host cytosol during the LS (66–69). Another possibility is that *Plasmodium* uses PVM-resident proteins to manipulate the host cell and redirect trafficking. Unfortunately, the composition of this host–pathogen interface during the LS is largely unknown. *Plasmodium* encodes its own SNARE proteins that regulate parasite membrane trafficking, and it would be interesting to investigate if any of these proteins are capable of forming a complex with host VAMP3 (70). This host–pathogen interaction has been reported in *Chlamydia*, where inclusion membrane proteins (Incs) are exported to the inclusion membrane for interactions with VAMP3 (71). Importantly, a *Plasmodium* SNARE protein and VAMP3 could only interact if they are in proximity to the PVM and topologically positioned toward the host cytosol, but predicting SNARE protein localization is challenging since they do not typically contain a signal peptide sequence. Alternatively, the parasite may utilize host SNARE machinery for vesicle fusion, a proposal supported by the recent aforementioned LS study (38). In that work, they document that lysosomal fusion is mediated by a two-phased recruitment of host SNARE proteins, where VAMP7 and Vti1B are recruited during invasion, and VAMP8 and Stx7 are recruited for development. Overall, a greater understanding of the LS exportome and PVM composition would enhance our understanding of these host-pathogen interactions. In particular, future studies on host and parasite SNARE localization will be critical for identifying the components of the VAMP3 ternary complex. Once SNARE complexes are formed, they require NSF for disassembly to allow VAMP recycling for future rounds of membrane fusion. Future studies could investigate a possible role for host NSF in vesicle recycling at the PVM.

Significantly, VAMP3 may be broadly utilized by intracellular pathogens as it has been implicated in *Chlamydia, Yersinia, Leishmania, Salmonella, Trypanosoma cruzi, Coxiella, Legionella,* and human cytomegalovirus (71–79). Though VAMP3 hijacking is conserved, it can serve different functions. During *Chlamydia* infection, *VAMP3* depletion decreases inclusion size but does not affect progeny production (71). Interestingly, *de novo* chlamydial protein synthesis is required for VAMP3 recruitment to the inclusion, indicating *Chlamydia* actively recruits VAMP3 vesicles during infection (71). In *Yersinia*, VAMP3 is only recruited in early infection, where it plays a vital role in the commitment toward replication in single-membrane LC3-positive vacuoles (73). Conversely, *Leishmania amazonensis,* but not *L. major*, has been shown to increase PV size without VAMP3, suggesting that VAMP3 vesicles are harmful and may contain antimicrobial cargo or that VAMP3 outcompetes alternative beneficial SNARE fusion events (74). This underscores VAMP3’s broad roles in pathogenic infections and the importance of understanding how it supports *Plasmodium* LS infection.

Here, we demonstrate that recruitment of the host protein CGA is dependent on host membrane trafficking systems, particularly exocytosis processes involving the SNARE protein VAMP3. Functional VAMP3 was important for proper LS development and survival, which could be through the delivery of essential nutrients (proteins or lipids). Using confocal and U-ExM, we show the recruitment of VAMP3 vesicles, which accumulate with NEM treatment. This provides evidence of host–parasite vesicle fusion in these pathogenic parasites. These insights open avenues for investigating the host–pathogen interface to reveal proteins and nutrients that are usurped by the parasite during LS development.

## MATERIALS AND METHODS

### Cell and parasite culture

HC-04, Huh7.5.1, and HepG2 cells (cells used for RNA-seq) were maintained in complete media (Dulbecco’s modified Eagle medium [DMEM], Gibco) supplemented with 10% fetal bovine serum (FBS), 0.29 mg/mL glutamine, 100 units of penicillin, and 100 μg/mL streptomycin in a standard tissue culture incubator at 37°C with 5% CO_2_. For all other studies, HepG2 cells (ATCC HB-8065, Duke Cell Culture Facility) were maintained in DMEM (Gibco) supplemented with 10% FBS (Millipore Sigma) and 1% antibiotic-antimycotic (Millipore Sigma) in a standard tissue culture incubator at 37°C with 5% CO_2_.

*Anopheles stephensi* mosquitoes infected with *P. berghei* ANKA stably expressing luciferase (*P. berghei*-Luc) and *P. berghei* ANKA stably expressing green fluorescent protein (*P. berghei*-GFP) were obtained from the Insectary Core Facility at New York University or from the SporoCore (University of Georgia, Athens). *P. berghei* sporozoites were isolated from mosquito salivary glands immediately before infection.

### Dual RNA-sequencing analysis and code availability

Raw reads published in LaMonte et al. were downloaded and reanalyzed (9). A count table was generated using Huh7.5.1, HC-04, and HepG2 cell lines at 0, 24, and 48 hpi for infected and uninfected cells as previously reported. Differentially expressed genes were obtained using DESeq2 with a multifactorial design for condition (uninfected or infected) and cell line at both 24 and 48 hpi (80).

### Infection of hepatocytes for qRT-PCR

Hepatocytes were seeded into 24-well plates (120,000 cells per well) 24 h before infection and then infected with *P. berghei*-GFP sporozoites obtained from freshly dissected and infected *A. stephensi* mosquitoes at a ratio of 0.3 sporozoites per seeded cell. Plates were centrifuged at 330 × *g* for 3 min, and then incubated at 37°C in 5% CO_2_ for 2 h to promote sporozoite invasion (3). After 2 h, the cells were washed with fresh complete DMEM supplemented with 12 μM 5-fluorocytosine (Cayman, cat #11635), 50 μg/mL gentamicin sulfate (Gibco, cat #15710072), 100 μg/mL neomycin trisulfate salt hydrate (Sigma-Aldrich, cat #72133), and returned to the incubator.

### Separation of infected and uninfected hepatocytes, RNA isolation, and gene expression quantification by qRT-PCR

HepG2, Huh7.5.1, and HC-04 cells, in addition to PHHs, were dissociated from plates at time zero (uninfected hepatocytes and sporozoites before infection), 24, and 48 hpi by the addition of 500 μL TrypLE Express (ThermoFisher), washed once using PBS (Gibco), and resuspended in FACS buffer (PBS supplemented with 1 mM EDTA, 25 mM HEPES, and 0.5% FBS), then passed through a 40 μm cell strainer prior to sorting (BD Falcon). Uninfected and infected cells were isolated by FACS using a BD Influx cell sorter, with GFP mean fluorescence intensity (MFI) used to determine infection status. The overall gating strategy shown in Fig. S1B is as previously reported (9), with total cells identified via forward scatter (FSC) vs side scatter (SSC) (P1 gate), individual cells identified via tandem gating of FSC-height vs FSC-width and SSC-height vs SSC-width (gates P2 and P3), and then GFP-positive and -negative cells were collected as indicated (gates P4 and P5, respectively). For RNA extraction, cells were directly sorted into Eppendorf tubes containing 600 μL Qiazol reagent (Qiagen), and total RNA was extracted using the Qiagen miRNeasy Mini kit according to the manufacturer’s instructions. Gene expression differences were measured using SYBR Green qRT-PCR with PerfeCTa SYBR Green FastMix (Quanta Bio), using the primers indicated in Table S3 and a Bio-Rad CFX96 qRT-PCR system (Bio-Rad). Changes were quantified according to the ΔΔ cycle threshold (Ct) method and normalized using the human beta-2-microglobulin (*B2M*) gene. If primers did not bind, Ct values were assigned as the limit of detection (Ct = 40 cycles).

### Genetic knockdown using siRNA reverse transfection

HepG2 cells (3,250/well) were reverse transfected with Lipofectamine RNAiMax (Invitrogen) diluted in OptiMEM (Gibco) with siRNAs (Qiagen; Table S4) in a pooled format with two siRNAs per gene added to cells in a 384-well plate (Corning). Controls included single siRNAs against the negative scramble control (siCTRL) and positive control (si*SCARB1*) (30). siRNAs were at a final concentration of 25 nM. After 48 h, half of the cells were infected with *P. berghei*-Luc (4,000/well) while the other half were extracted for RNA isolation. After an additional 48 h, HepG2 and *P. berghei* viability were measured via CellTiter-Fluor (Promega) and Bright-Glo (Promega), respectively, using an Envision plate reader (PerkinElmer). Data were normalized to the non-targeting scramble siRNA (siCTRL) control. Samples were evaluated in six technical replicates across 2 or 3 biological replicates.

### RNA extraction and qRT-PCR analysis of knockdown cells

RNA was extracted from cells using the Zymo microRNA prep kit (Zymo Research). RNA was normalized to 500–750 ng for reverse transcription using GoScript reverse transcriptase (Promega) to yield cDNA per the manufacturer’s instructions. Relative mRNA expression was assessed with 2× SYBR Green Fast qPCR (Abclonal) in a 384-well plate using a LightCycler 480 Instrument II (Roche Diagnostics). Gene-specific primers are indicated in Table S5. The Ct values for each gene were normalized to the human *18S* housekeeping gene Ct values. Data were further normalized to the negative control (siCTRL), and the relative amount of mRNA was calculated as 2^−ΔΔCt^ {ΔΔCt = [Ct − Ct(18S)] − [Ct(siCTRL) − Ct(siCTRL 18S)]}. Samples were evaluated in technical triplicate across 2–3 biological replicates.

### Purification of *P. berghei* MSP1 antibody

The murine hybridoma cell line L1.6 anti-*P*. *berghei* MSP1 was obtained from BEI Resources (MRA-667, contributed by Mark F. Wiser). Cells were cultured in Advanced RPMI 1640 (Gibco, 12633012) with 4 mM L-glutamine (Gibco, 25030081) and gentamicin (50 μg/mL) supplementation. Hybridoma cells were grown until confluent in a T75 flask, and the supernatant was collected and incubated with Protein G Sepharose Fast Flow beads (Millipore Sigma, P3296) overnight at 4°C. The beads were washed with PBS, and the antibodies were eluted with 100 mM glycine, pH 2.5. Collected fractions were neutralized with 1 M TRIS, pH 9.5, and a small sample was separated on an SDS-PAGE gel. The fractions containing the antibody were pooled together and concentrated. The purified MSP1 antibody was diluted to a final concentration of 2.0 mg/mL containing 0.02% sodium azide and stored at −20°C until use.

### Fixed immunofluorescence microscopy staining

HepG2 cells (5,500 cells/well) were seeded on glass 384-well plates (Cellvis-P28415HN) and allowed to recover overnight before infection with *P. berghei-*Luc sporozoites (4,000/well). At the indicated times, cells were fixed in 4% paraformaldehyde (PFA) for 10 min and then washed with PBS. For CGA staining, cells were permeabilized with 0.1% Triton X-100 in PBS for 10 min, washed with PBS, and incubated with blocking buffer 1 (0.05% Tween 20 in PBS [PBST] with 3% BSA) for 1 h. The primary antibodies rabbit anti-HsCGA (LS-Bio, cat# LS-C293331-100, 1:50) and goat anti-PbUIS4 (antibodies.com, cat# A121573, 1:1,000) were diluted in blocking buffer 1 and incubated overnight at 4°C. After washing with PBST, cells were incubated with Alexa Fluor-conjugated antibodies, donkey anti-goat 488 (Invitrogen, cat# A11055, 1:400) or donkey anti-rabbit 568 (Invitrogen, cat# A10042, 1:400), diluted in blocking buffer 1 for 1 h at RT. For HsLC3 and HsVAMP3 staining, cells were permeabilized and incubated with blocking buffer 2 (0.1% Triton X-100 with 3% BSA) for 1 h at RT. Subsequently, primary antibodies mouse anti-HsLC3 (MBL, cat# M152-3, 1:200) or rabbit anti-VAMP3 (Abcam, cat# ab200657, 1:400) and goat anti-PbUIS4 (antibodies.com, cat# A121573, 1:1,000) were diluted in blocking buffer 2 and incubated for 1 h at RT (HsLC3) or overnight at 4°C (HsVAMP3). After washing with PBST, cells were incubated with Alexa Fluor-conjugated antibodies, donkey anti-goat 488 (Invitrogen, cat# A11055, 1:400) or donkey anti-mouse 568 (Invitrogen, cat# A10037, 1:400), diluted in blocking buffer 2 for 1 h at RT. For PbMSP1 staining, cells were permeabilized with 0.1% Triton X-100 in PBS for 10 min, washed with PBS, and incubated with blocking buffer 1 for 1 h. The primary antibodies mouse anti-MSP1 (1:200) and goat anti-PbUIS4 (1:1,000) were diluted in blocking buffer 1 and incubated overnight at 4°C. After washing with PBST, cells were incubated with Alexa Fluor-conjugated antibodies, donkey anti-goat 488 (1:400) or donkey anti-mouse 568 (Invitrogen, cat# A10037, 1:400), diluted in blocking buffer 1 for 1 h at RT. After antibody staining, cells were washed in PBST, and nuclei were stained with DAPI (0.5 μg/mL) for 10 min at RT. Cells were washed with PBS before imaging.

*P. falciparum*-infected PHHs were incubated with PbUIS4 (1:500) and rabbit anti-HsVAMP3 (Synaptic Systems, cat# 104103, 1:200) in blocking buffer 3 (1% BSA in 0.03% Triton X-100) for 1 h at RT. After washing with PBS, cells were incubated with Alexa Fluor-conjugated antibodies, donkey anti-goat 488 (1:400) and donkey anti-rabbit CF568 (1:400), diluted in blocking buffer 3 for 1 h at RT. Cells were stained with DAPI as above and washed before imaging.

### *P. falciparum* liver stage infections for immunofluorescence

*P. falciparum* LS infections were completed following previously published methods with slight modifications (81). *P. falciparum* isolates were adapted to laboratory culture conditions and cloned to assess compatibility to infect *A. stephensi* mosquitoes. *P. falciparum* isolates and clone Cam5 (Cambodia) were cultured to induce gametocytogenesis with cryopreserved RBCs (Interstate Blood Bank, North Carolina, USA). Briefly, asexual and gametocyte cultures were maintained at 38°C in an incubator with 5% O_2_ and 5% CO_2_ (Panasonic). Cultures received a daily medium change with RPMI 1640 + L-glutamine + 25 mM HEPES + 50 µg/mL hypoxanthine (KD Medical) and 5% heat-inactivated pooled human serum (Interstate Blood Bank, NC, USA). The asexual culture was kept between 0.5% and 5% parasitemia, and gametocyte culture was seeded at 0.5% parasitemia with cryopreserved O^+^ human RBCs (5% hematocrit). The gametocyte culture was assessed for exflagellation using a hemocytometer (INCYTO) to count the number of exflagellation centers between 12 and 18 days post-seeding. Once deemed infectious for 12–18 days post-seeding, the culture was offered to 150–250 blood-thirsty 3- to 7-day-old female *A. stephensi* mosquitoes as described in a previous publication (81). Infected mosquitoes were kept at 24°C ± 5°C, 80% ± 5% relative humidity, and under a 12-h day/night photoperiod schedule (Percival Scientific) with 5% (wt/vol) dextrose and 0.05% (wt/vol) para-aminobenzoic acid (Thermo Fisher). Mosquito oocysts were checked at 10 dpi, and salivary glands were collected between 18 and 20 dpi.

*P. falciparum* infection of PHH followed published protocols (82). Briefly, cryopreserved PHHs from donor TWJ (BioIVT) were thawed 2 days prior to infection and seeded at 18,000 cells per well in a 384-well collagen-coated plate (Greiner). Hepatocyte culture was maintained with daily medium changes with customized InVitroGro HI medium (BioIVT) and supplemented with 5% human serum (Interstate Blood Bank), 1:100 dilution of penicillin/streptomycin/neomycin antibiotic mixture (Gibco), and 1:1,000 dilution of gentamicin (stock concentration: 10 mg/mL, Gibco). The salivary glands of *P. falciparum-*infected *A. stephensi* mosquitoes were collected between 18 and 20 dpi, and 24,000 sporozoites were inoculated into the hepatocyte culture. The plate was centrifuged at RT at 250 × *g* for 5 min. Hepatocyte cultures were kept at 38°C with 5% CO_2_ (Panasonic). Infected hepatocytes were destructively sampled at 4 dpi, and the cultures were fixed with 4% paraformaldehyde (Alfa Aesar) for 45 min at RT.

### Confocal microscopy

Images were acquired using a Zeiss Airyscan 880 inverted confocal microscope with a Märzhäuser linearly encoded *x*, *y* stage and a 63× 1.40 NA oil-immersion plan Apochromat objective (CGA recruitment [Fig. 1 and 2]) and a 40× 1.20 NA water-immersion C Apochromat objective (expansion microscopy [Fig. 5 and 6]). Laser illumination was via Argon for 488 nm and diode for 405 nm and 561 nm. The fluorescence signal was collected with two photomultiplier tubes and one GaAsP detector in the following emission ranges: 415–487 nm—DAPI, 490–570 nm—Alexa Fluor 488 (GaAsP), 570–633 nm—Alexa Fluor 568, CF568 (GaAsP). Images were acquired sequentially by line scanning bidirectionally with line averaging of 4 and a pinhole calculated to be 1 airy unit for green using Zeiss Zen software (version 2.3) and saved as Carl Zeiss Image files.

Images for VAMP3 and LC3 association studies (Fig. 3 to 6) were acquired using a Leica MICA inverted confocal microscope with a Leica Scanning Stage and a 63 × 1.40 NA oil-immersion plan Apochromat objective (HC PL APO CS2). Laser illumination was via diodes specific for 405 nm—DAPI, 488 nm—Alexa Fluor 488, 561 nm—Alexa Fluor 568. Fluorescent signals were detected using 4 multi-pixel HyD FS silicon photomultiplier arrays in the indicated ranges. Images were acquired sequentially by line scanning bidirectionally with line averaging of 4 and a pinhole calculated to be 1 airy unit for each channel using Leica LASX software (version 1.4.7) and saved as Leica Object Files.

### Image analysis

FIJI was used to crop, add scale bars, and save images as jpg and tiff files for each channel and merged (83). For PV size determination of membrane trafficking member knockdown (Fig. 2), the PVM, excluding membrane clusters, was traced and measured (FIJI) in images acquired at the middle focal plane of the vacuole. CGA recruitment was determined visually by two scientists individually and scored as positive or negative for recruitment as determined by overlapping signal between green and red channels. Images with different scores were discussed and determined as a group. Samples were evaluated as >2 technical replicates that included >50 PVs across two biological replicates.

Aivia 14 software was used to determine PV size in *VAMP3* knockdown and VAMP3 association experiments (Fig. 3 to 6). Images were imported, and PVs were recognized using the Cellpose algorithm using the green (UIS4) channel with the following constraints: 24 hpi (software model 3, average diameter set to 4 μm, probability set to 40), 36 hpi (software model 3, average diameter set to 6 μm, probability set to 60), and 48 and 58 hpi (software model 3, average diameter set to 8 μm, probability set to 70). To validate accurate PV detection, images were manually confirmed, and shapes were edited using the object modifier tool when needed. For MSP1 mean gray value quantification, the average intensity of the red channel was calculated for each PV object.

Aivia 14 software was also used to analyze images for both association and colocalization. For LC3 and VAMP3 recruitment analysis, images were imported, and the green (UIS4) and red (LC3 or VAMP3) signals were thresholded using Cell Count to create objects. Once the signals were computed as objects, overlap between UIS4 and LC3 or VAMP3 was calculated. Recruitment was scored as positive if the overlap between UIS4 and LC3/VAMP3 was >1 µm^2^. An overlap coefficient was calculated by dividing the overlap area by the total UIS4 area. Samples were evaluated for > 100 PVs at each time point across three biological replicates and >2 technical wells.

### Relative infection rate assay

For determining the number of parasites per well, cells were stained with DAPI and anti-UIS4 (as above), and whole-well stitched images were acquired with widefield using the 20× objective (HC PL APO 20x/0.75 CS2) with 7 *z*-planes on the Leica MICA system. Maximum projection images were used to count the number of parasites manually in the LASX software.

### Pre-gelation staining expansion microscopy

Glass coverslips (5 mm, Electron Microscopy Sciences) were sterilized in ethanol and dried. HepG2 cells (20,000/well) were seeded in 96-well plates containing the 5 mm coverslips. After ~24 h, cells were infected with *P. berghei*-Luc (16,000 sporozoites/well) and fixed in 4% PFA at RT for 10 min at 48 hpi. Coverslips were then processed for microscopy as above for primary antibodies. After primary antibody incubation, coverslips were washed in PBST, and secondary antibodies (donkey anti-goat Alexa Fluor Plus 488 [Invitrogen, cat# A3281] and donkey anti-rabbit CF568 [Biotium, cat# 20098]) were added at 1:200 for 1 h at RT. Pre-gelation staining was based on the previously reported protocols published by Gambarotto et al. and Atchou et al. (46, 84). The day before expansion, a monomer solution was prepared containing 23% (wt/vol) sodium acrylate (solubilized in water, Sigma-Aldrich), 10% acrylamide (Sigma-Aldrich), 0.1% N,N′-methylenbisacrylamide (Sigma-Aldrich) in PBS. After staining, samples were anchored with 0.7% formaldehyde (Sigma-Aldrich) and 1% acrylamide (Sigma-Aldrich) in PBS for 1.5 h at 37°C. Samples were washed with PBS before the addition of 0.5% ammonium persulfate (solubilized in H_2_O, ThermoFisher) and 0.5% tetramethylethylenediamine (ThermoFisher) on ice to activate the monomer solution as the free radical accelerator and initiator, respectively. For 5 mm coverslips, 6.25 μL of total gelation solution was pipetted onto parafilm-coated glass slides. The samples were incubated on ice for 5 min, transferred to a moisture-controlled chamber, and incubated at 37°C for 30 min to finish the gelation process. Gels (still attached to coverslips) were transferred to an Eppendorf tube containing denaturation buffer (50 mM Tris, pH 9, 200 mM NaCl, and 200 mM SDS in ultrapure water) and then incubated at 95°C for 30 min. Gels were washed briefly with PBS, transferred to a 6-well plate, and incubated with DAPI (1:1,000 in PBS) for 1 h at RT with gentle agitation. The gels were washed with PBS before placement in ultrapure water for 30 min with gentle agitation (2×) and an overnight exchange in water on the bench at RT. Gels were cut and mounted on poly-D-lysine (Gibco)-coated 2-well chamber slides (Ibidi).

### Atovaquone and cabamiquine treatment

HepG2 cells were seeded and infected with *P. berghei*-Luc as above. At 24 hpi, cells were either fixed or treated with DMSO, atovaquone (20 nM), or cabamiquine (10 nM) from 24 to 48 hpi. At 48 hpi, cells were fixed in 4% PFA for 10 min and then stained with DAPI, anti-PbUIS4, and anti-HsVAMP3 or anti-HSLC3 and imaged (described above). LC3 recruitment was assessed as a binary for >50% PVM coverage, and VAMP3 recruitment was quantified as above.

### NEM treatment

HepG2 cells were seeded and infected with *P. berghei*-Luc as above. At 47.5 hpi, cells were treated with DMSO or 250 μM NEM (Thermo Scientific) for 30 min at 37°C and subsequently fixed in 4% PFA for 10 min. Cells were stained as above with DAPI, anti-PbUIS4, and anti-HsVAMP3 and imaged. VAMP3 recruitment was quantified as above.

### Statistical analysis

All data were analyzed using GraphPad Prism 10 with the statistical tests described in the figure legends.

## SUPPLEMENTAL MATERIAL

Supplemental figures and tables mbio.01430-26-s0001.pdf Tables S2-S5; Figures S1-S14;

Table S1 mbio.01430-26-s0002.xlsx DEG at 24 and 48 hpi.

ASM does not own the copyrights to Supplemental Material that may be linked to, or accessed through, an article. The authors have granted ASM a non-exclusive, world-wide license to publish the Supplemental Material files. Please contact the corresponding author directly for reuse.
