# Supplementary material for: VAMP3 vesicles support host protein recruitment and Plasmodium liver stage development
Source: mBio. 2026 Jul 21;17(8):e01430-26. doi: 10.1128/mbio.01430-26 (PMC13463841; doi:10.1128/mbio.01430-26)
Supplement: Supplementary file 1 [file mbio.01430-26-s0001.pdf]

VAMP3 Vesicles Support Host Protein Recruitment and *Plasmodium* Liver Stage Development

Michael E. Chirgwin[1], Chelsea Gan[1], Karla P. Godinez-Macias[2], Kaitlyn Choe[1], Justine C. Shiau[3], Jaeson Calla[2,4], Dennis E. Kyle[3], Elizabeth A. Winzeler[2,5], Emily R. Derbyshire[1,6]

Affiliations:
[1]Department of Chemistry, Duke University, Durham, North Carolina, USA
[2]Department of Pediatrics, School of Medicine, University of California, San Diego, La Jolla, USA
[3]Center for Tropical and Emerging Global Diseases, University of Georgia, Athens, GA, USA
[4]Doctoral Programme in Health Sciences, Faculty of Medicine, Universidad Nacional Mayor de San Marcos, Lima, Peru
[5]Skaggs School of Pharmacy and Pharmaceutical Sciences, University of California, San Diego, La Jolla, USA
[6]Department of Molecular Genetics and Microbiology, Duke University School of Medicine, Durham, North Carolina, USA

To whom correspondence should be addressed: emily.derbyshire@duke.edu (E.R.D.)

Other supplemental materials for this manuscript: Table S1

Table S2. Membrane trafficking proteins chosen for this study

Gene	Gene ID	Cellular Process
GBF1	8729	Golgi
EEA1	8411	Early endosomes
PDCD6IP	10015	Endosomes/Lysosomes
ATP6V0A1	535	Endosomes/Lysosomes
TSG101	7251	Endosomes/Lysosomes
VPS4A	27183	Endosomes/Lysosomes
SNX1	6642	Endosomes/Lysosomes
CAV2	858	Endocytosis
DNM2	1785	Endocytosis
HIP1	3092	Endocytosis
ITSN1	6453	Endocytosis
SYNJ2	8871	Endocytosis
EXOSC7	23016	Exosomes
VAMP3	9341	Exocytosis
VAMP4	8674	Exocytosis
CLTA	1211	Coat protein
SEC24C	9632	Coat protein
ARF1	375	Coat protein
NSF	4905	Vesicle fusion
CFL1	1072	Vesicle fusion
WASF1	8936	Actin polymerization

Table S3. RT-qPCR primers used in this study to confirm upregulation genes.

Target	Primer direction	Sequence (5'→ 3')
NR4A3	Forward	TCCGCTCCTCCTACACTCTC
	Reverse	GACGCAGGGCATATCTGGAG
MUC13	Forward	ATGGCTGTAACCAGACTGCG
	Reverse	CTTGAGACTGGAAGCAACGC
CGA	Forward	CCCTCTTCGGATCCACAGTC
	Reverse	ATCCATGGCGCTCCTTTCTC
SDHA	Forward	TTGATTCCTCCCTGTGCTGC
	Reverse	GCATTTGGCCTTTCTGAGGC
TAS2R4	Forward	GAATCCCCAGACGGAAGCTC
	Reverse	GTCCCCATATCCATCCCTGC
B2M	Forward	AAGATGAGTATGCCTGCCGT
	Reverse	TGCTTACATGTCTCGATCCCAC

Table S4. siRNAs used in this study.

Gene	Catalog number	Sequence (5'→ 3')
Allstar scramble	1027280	Proprietary
Hs_SCARB1	D-010592-03-0010	ACTGACCGGACGTGGGATT
Hs_Arf1_1	SI00299250	ACGTGGAAACCGTGGAGTACA
Hs_Arf1_8	SI02654470	CACCATAGGCTTCAACGTGGA
Hs_ATP6V0A1_5	SI04199489	TACCAATAAACTGACGTTCTT
Hs_ATP6V0A1_6	SI04249707	CCGATTATGGACACCGGTGAA
Hs_CAV2_5	SI00299649	AACTACGCACTCCTTTGACAA
Hs_CAV2_9	SI02625644	CAGACTTCTCGGAATTCATAA
Hs_CFL1_1	SI00078190	CAGTAAGGGACCTTCGATTAA
Hs_CFL1_6	SI02654134	CAAGCATGAATTGCAAGCAAA
Hs_CGA_4	SI00344134	CAGAATGCACGCTACAGGAAA
Hs_CGA_5	SI02777327	CGCCATGGATTACTACAGAAA
Hs_CLTA_1	SI00299866	AGCCTGAAAGTATCCGTAAAT
Hs_CLTA_9	SI04326483	ACGGGTAGGGCTTCCGCTTTA
Hs_DNM2_8	SI02654687	CTGCAGCTCATCTTCTCAAAA
Hs_DNM2_9	SI03233671	TCGCAACCTGGTGGACTCATA
Hs_EEA1_6	SI04223800	AAGCTAAGTTGCATTCCGAAA
Hs_EEA1_9	SI05037452	CTCCCACGCATTGTCCATTTA
Hs_EXOSC7_10	SI04953949	TCGAAGGACATTGAATTGTCA
Hs_EXOSC7_9	SI04953942	CTGCATTGTCACTCTGTGCAA
Hs_GBF1_4	SI00425425	AAGGAAGACCTTACTGATCTA
Hs_GBF1_5	SI03158750	CACAAGGTTACCTCAGTTTAA
Hs_HIP1_1	SI00075838	CAGGAACTTGCCACAAGCCAA
Hs_HIP1_4	SI00075859	CAGGTTAGGGTGCTAGAGCTA
Hs_ITSN1_6	SI04161136	CAAGAACGAAAGATCATAGAA
Hs_ITSN1_9	SI04346419	CTGGTCCAGGGTCACAACTAA
Hs_NSF_12	SI04255608	AGGCAGACTTTCTACATGCAA
Hs_NSF_6	SI03019303	AAGGAATGCAATAAAGAGTAA
Hs_PDCD6IP_1	SI00681219	GAGGTACTTTATACTAACATA
Hs_PDCD6IP_6	SI03119354	TGGAAGGATGCTTTCGATAAA
Hs_SEC24C_3	SI00713965	CCCGTTGAGAGTACTACCGAA
Hs_SEC24C_8	SI04253564	CTGGAGGTTAGCAGTCAAGTT
Hs_SNX1_1	SI00047775	CGCGGTGGTCAGTAAACATCA
Hs_SNX1_7	SI03104003	GAGGGCCGCTTTAGAAAGGTA
Hs_SYNJ2_5	SI03044125	AGCGTCAGTCCGAATTCACAA
Hs_SYNJ2_6	SI03113187	TCCAACCGGTTTAATGGTGAA
Hs_TSG101_3	SI00087059	ACTGTCAATGTTATTACTCTA
Hs_TSG101_8	SI02664529	CAGCTGAGGGCACTAATGCAA
Hs_VAMP3_2	SI00759234	CAGGCGCTTCTCAATTTGAAA
Hs_VAMP3_6	SI04216443	TCCCTAGAAGAGAACCCTCTA
Hs_VAMP4_11	SI05471802	CTCATTGCCGGTATACTCTAA
Hs_VAMP4_7	SI04131134	AACATGGTATGGCATCATAAA
Hs_VPS4A_6	SI04227636	AACGGAGTTCTTGGTCCAGAT
Hs_VPS4A_8	SI04349086	AAGGCCAAGGAGAGCATTCGA
Hs_WASF1_3	SI00057939	CAAGAACGTGTGGACCGTTTA
Hs_WASF1_8	SI05064423	TTAGCATACATTTCAAACTAA

Table S5. RT-qPCR primers used in this study to confirm knockdown.

Target	Primer direction	Sequence (5'→ 3')
18S rRNA	Forward	GGCCCTGTAATTGGAATGAGTC
	Reverse	CCAAGATCCAACTACGAGCTT
SCARB1	Forward	TCTCTCCGTCTACTTCTTTGACG
	Reverse	AACTGGAAGGTGCGGTACTC
ARF1	Forward	CAATGACAGAGAGCGTGTGA
	Reverse	GAGGTCCTGCTTGTTGGC
ATP6V0A1	Forward	TTACGGACGCAAGCACGTC
	Reverse	AAAAGCTGGGCCAGTGTCAT
CAV2	Forward	AGCTCTTCATGGACGACGAC
	Reverse	AAAGGAGTGCGTAGTCACCG
CFL1	Forward	GGAAGACTCCGTTACCCAGC
	Reverse	CGTTTAGTAGGAGCCGCACA
CGA	Forward	TGTGCAGGATTGCCCAGAAT
	Reverse	TGGACCTTAGTGGAGTGGGA
CLTA	Forward	TGGAAGCCCTTGATGCCAAT
	Reverse	AATCACGTCAGCGAAGGGTT
DNM2	Forward	AGCAGGTCGTCAAGCTGAAA
	Reverse	CTCCCGTTCCCGGATGTAAG
EEA1	Forward	GCTCTTCAGAGGGTTTCATATGTC
	Reverse	CTTGGACCTCTTGTCTGAGCA
EXOSC7	Forward	CTTGGTGGGAGTGAAAGCAGA
	Reverse	CCCGAGGACTAATGCAGAGG
GBF1	Forward	CTCCCAGAAAGAAGGCACAG
	Reverse	GCTACAGGGGCTGACTCAAG
HIP1	Forward	CCGAGGGCCCCTGACTC
	Reverse	ACAGCCACTTCCTGCGTATT
ITSN1	Forward	CGAAGGAGGTAGAGAAGAGTGGA
	Reverse	CCGCTCGGGATTCTTCCT
NSF	Forward	CCTGCAAGTGACGAGAGGAG
	Reverse	GGTGTGCGGTCACTGTTCTT
PDCD6IP	Forward	CCTTAAGTCGAGAGCCGACC
	Reverse	CAGGGAACACCTCCTGGAAA
SEC24C	Forward	CCTGGGAGCCTTGACGTTAG
	Reverse	CCCCAGCCAGCATTCAGATT
SNX1	Forward	ATGGCTTCATTGTCCCTCCG
	Reverse	ACCTTTCTAAAGCGGCCCTC
SYNJ2	Forward	CAACTTCTTCGACGGGGTGA
	Reverse	TTCCTCCGTTCACGTTCCAG
TSG101	Forward	GCCAGCTCAAGAAAATGGTGT
	Reverse	AGGCACAGGGATTGTTCCAG
VAMP3	Forward	GAGTTAGACGACCGTGCAGA
	Reverse	ATCCCGATTGCCCACATCTTG
VAMP4	Forward	ATTGCTGCCTGGGATATGGG
	Reverse	GGTAGGCCTGGCATCAGTTT
VPS4A	Forward	GAAACACGGCAAGAAGCCAG
	Reverse	TTGGGCTTCTCCATCACGAC
WASF1	Forward	ACTCACTTTCTGCCTTGCCA
	Reverse	TGTTCTGCCTGTAGCTGGTG

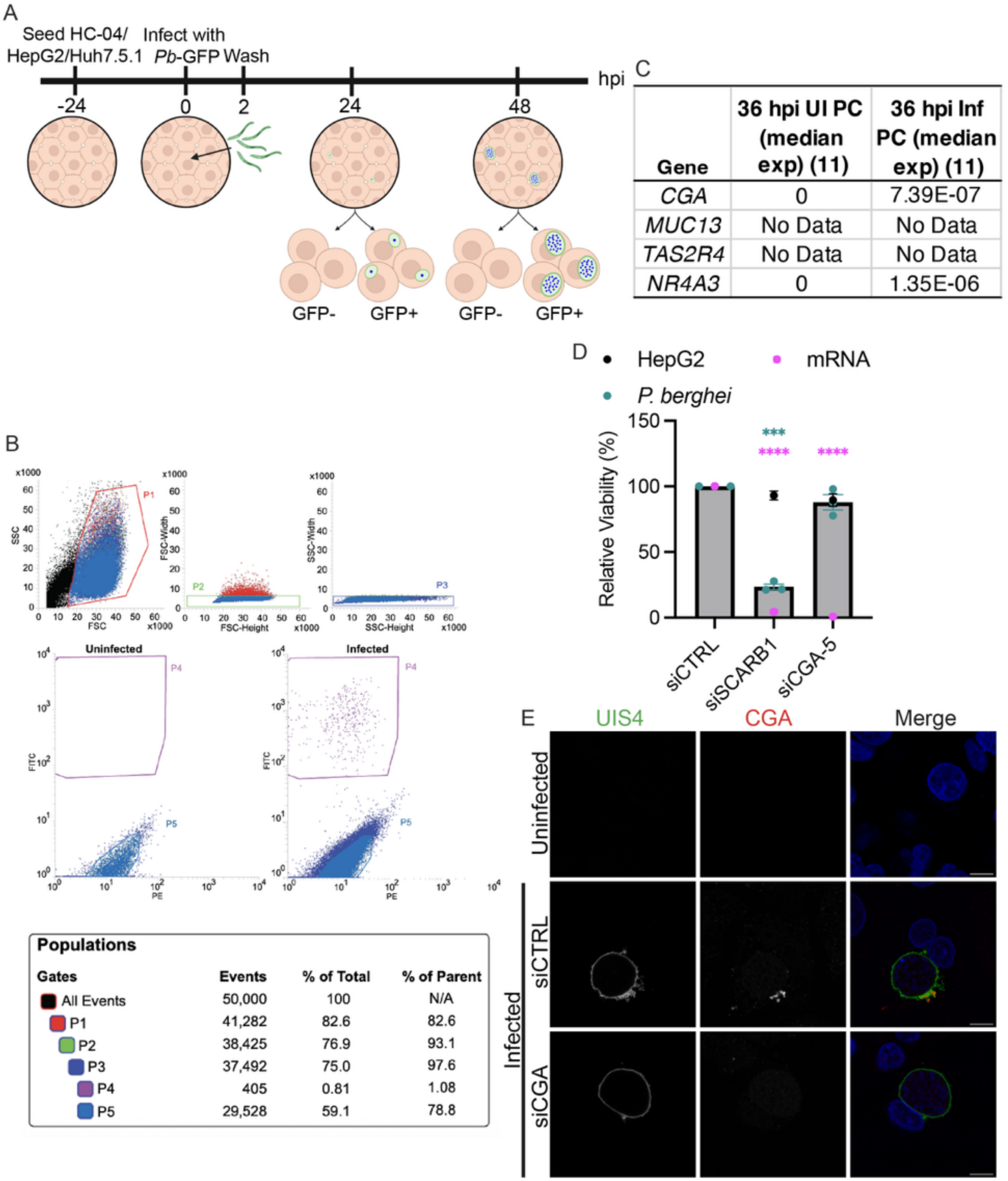

Gene	36 hpi UI PC (median exp) (11)	36 hpi Inf PC (median exp) (11)
CGA	0	7.39E-07
MUC13	No Data	No Data
TAS2R4	No Data	No Data
NR4A3	0	1.35E-06

Figure S1. CGA is upregulated at the protein level. A) Schematic representation of *P. berghei* infections and sorting by FACs. B) Representative FACS plots illustrating the gating strategy used to obtain *P. berghei*-infected hepatocytes. Gates for P1 (FSC vs SSC), P2 (FSC-height vs FSC-Width), P3 (SSC-Height vs SSC Width), P4 (GFP Positive in FITC vs PE) and P5 (GFP negative in FITC vs PE) are indicated, as well as number (out of 50,000 total events) and percentage of events. C) Uninfected (UI) and infected (Inf) pericentral (PC) lobule zone hepatocyte median

expression of the indicated gene at 36 hpi from the scRNA-seq dataset by Afriat et al. (11) D-E) HepG2 cells were reverse transfected with siCTRL, siSCARB1 (positive control) or siCGA (25 nM) for 48 h before infection with *P. berghei*-Luc. D) HepG2 viability (black circles) and parasite load (bar) were measured 48 hpi. Relative mRNA levels (pink circles) for *SCARB1* and *CGA* were determined using qRT-PCR. Data was normalized to the negative control and shown as mean ± SEM. $n = 3$ biological replicates. *p*-values display Welch's two-tailed t-test compared to siCTRL. *p*-values display two-tailed t-test compared to siCTRL. ***$p < 0.01$, ****$p < 0.001$. E) Representative confocal microscopy images of cells fixed at 48 hpi and stained for anti-PbUIS4, anti-HsCGA, and DAPI. CGA staining is not observed in uninfected cells or in cells with siCGA treatment before *P. berghei*-infection. Scale bar is 10 µm.

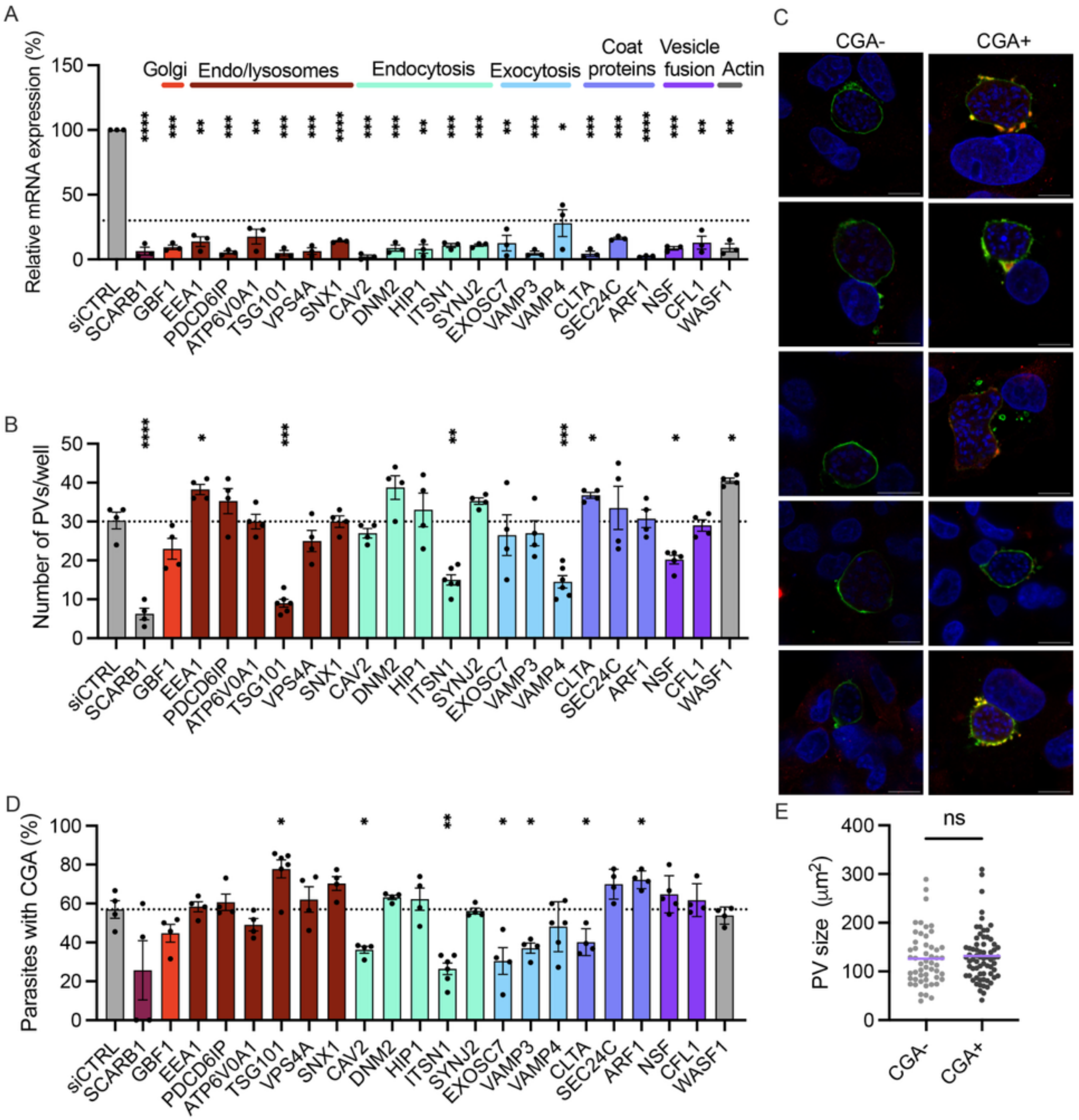

Figure S2. Membrane trafficking gene knockdown assays. A-D) HepG2 cells were reverse transfected with siRNAs targeting human trafficking members involved in Golgi function (light red), endosomes/lysosomes (dark red), endocytosis (green), exocytosis (blue), coat proteins (light purple), vesicle function (dark purple) and actin (grey). A) Relative mRNA levels of HepG2 cells in Figure 2 was determined with qRT-PCR. Data was normalized to the negative control *18S* and shown as mean ± SEM. *n* = 3 biological replicates. *p*-values display Welch's two-tailed t-test compared to siCTRL. B) The number of PVs per well were counted and plotted in each condition. Data was normalized to the negative control and shown as mean ± SEM. *n* = 2-4 wells for 2 biological replicates. *p*-values display Welch's two-tailed t-test compared to siCTRL. C) Merged confocal microscopy images of *P. berghei*-infected HepG2 cells stained with DAPI (blue), anti-

PbUIS4 (green) and anti-HsCGA (red). Additional images with (right) or without CGA recruitment (left) to the PV at 48 hpi are shown. Scale bar is 10 µm. D) For each well assessed for CGA recruitment, the percent PVs recruiting CGA was plotted, showing consistency between technical and biological replicates. Data was normalized to the negative control and shown as mean ± SEM. *n* = 2-4 wells for 2 biological replicates. *p*-values display Welch's two-tailed t-test compared to siCTRL. E) PV size for PVs with (CGA+) and without CGA (CGA-) recruitment shows no correlation. *p*-value display Welch's two-tailed t-test compared to CGA-. (A-E) not significant (ns) > 0.05, *$p < 0.05$; **$p < 0.01$; ***$p < 0.005$; ****$p < 0.001$.

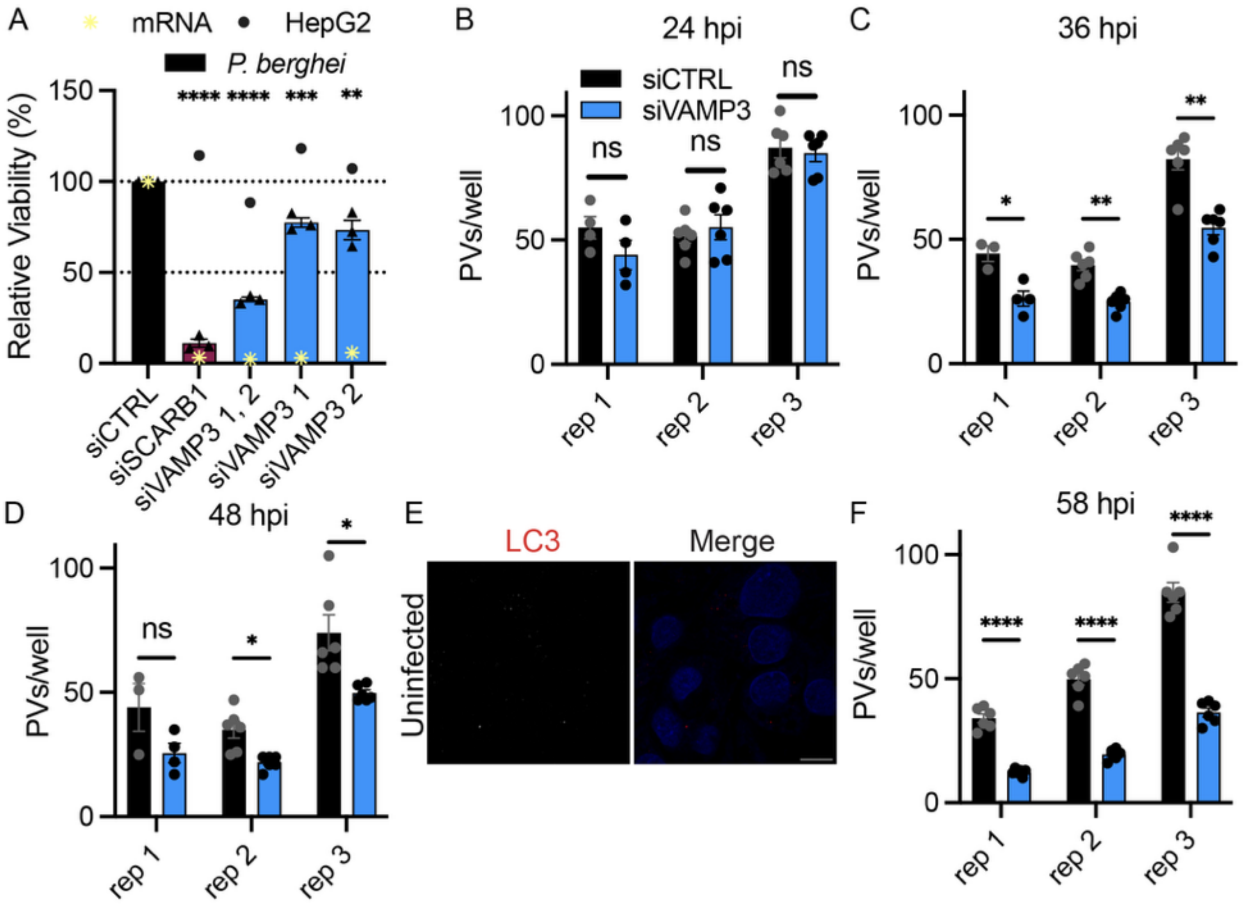

Figure S3. Impact of *VAMP3* depletion on *P. berghei* LS. A) HepG2 cells were reverse transfected with siCTRL or individual siRNAs targeting *SCARB1* (positive control) or *VAMP3* (25 nM) for 48 h before infection with *P. berghei*-Luc. Cell viability (black circles) and parasite load (bar) were measured at 48 hpi. Relative mRNA levels (yellow star) for *SCARB1* and *VAMP3* were determined using qRT-PCR. Data represents mean ± SEM. *n* = 3 biological replicates. B-D) HepG2 cells were reverse transfected with siCTRL or pooled siVAMP3 (25 nM) for 48 h before infection with *P. berghei*-Luc. Cells were fixed at B) 24, C) 36, and D) 48 and stained for anti-PbUIS4 and DAPI. The number of PVs per well was quantified where each dot represents one well. *n* = 3 biological replicates analyzing 3-6 wells. *p*-values display Welch's two-tailed t-test compared to siCTRL. E) Representative confocal microscopy images of LC3 staining in uninfected HepG2 cells. Cells stained with anti-HsLC3 (red) and DAPI. No LC3 staining is observed, as expected. Scale bar is 10 µm. F) HepG2 cells were reverse transfected with siCTRL or pooled siVAMP3 (25 nM) for 48 h before infection with *P. berghei*-Luc. Cells were fixed at 58 hpi and stained for anti-PbUIS4 and DAPI. The number of PVs per well was quantified where each dot represents one well. *n* = 3 biological replicates analyzing 6 wells. *p*-values display Welch's two-tailed t-test compared to siCTRL. not significant (ns) > 0.05, *$p < 0.05$; **$p < 0.01$; ***$p < 0.005$; ****$p < 0.001$.

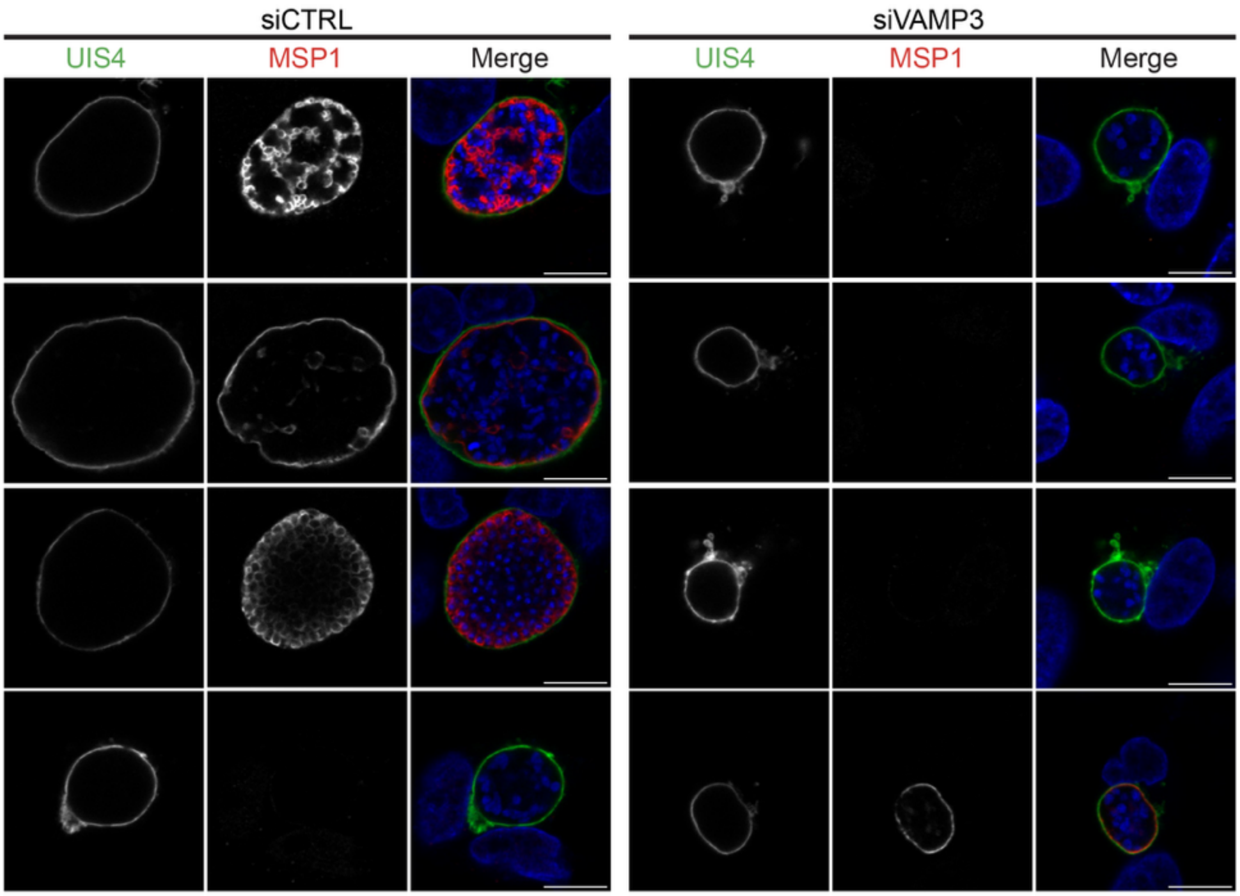

Figure S4. Parasites in *VAMP3* deficient cells express less MSP1. Additional confocal microscopy images of HepG2 cells reverse transfected with siCTRL or siVAMP3, infected with *P. berghei*-Luc, and fixed at 58 hpi. Cells were stained with anti-PbUIS4 (green), anti-PbMSP1 (red), and DAPI (blue). Scale bar is 10 µm.

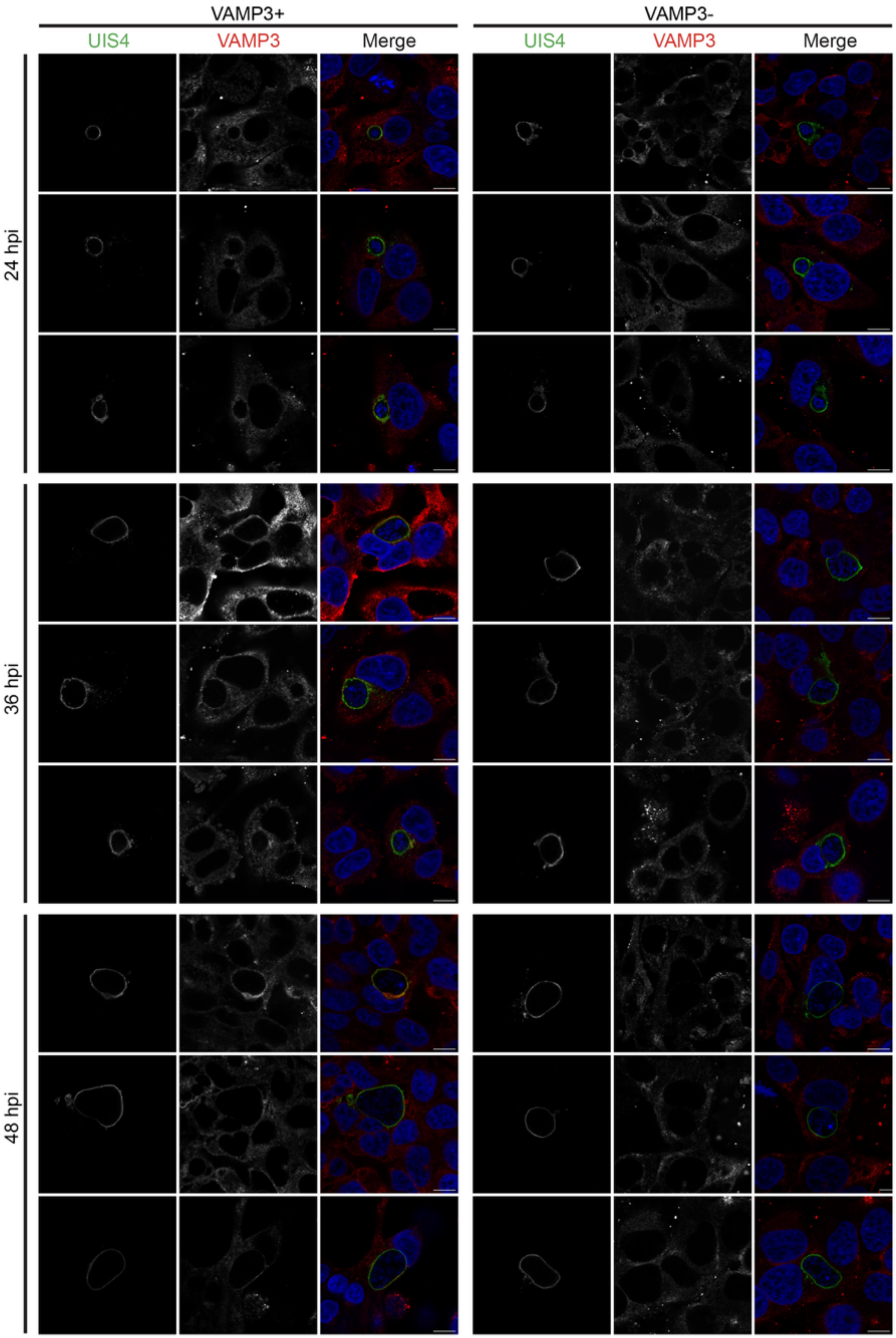

VAMP3+
VAMP3-
UIS4
VAMP3
Merge
UIS4
VAMP3
Merge
24 hpi
36 hpi
48 hpi

Figure S5. VAMP3 associates with the PVM. Additional confocal microscopy images of *P. berghei*-infected HepG2 cells fixed at 24, 36 or 48 hpi. Cells were stained with anti-PbUIS4 (green), anti-HsVAMP3 (red) and DAPI (blue). Scale bar is 10 µm.

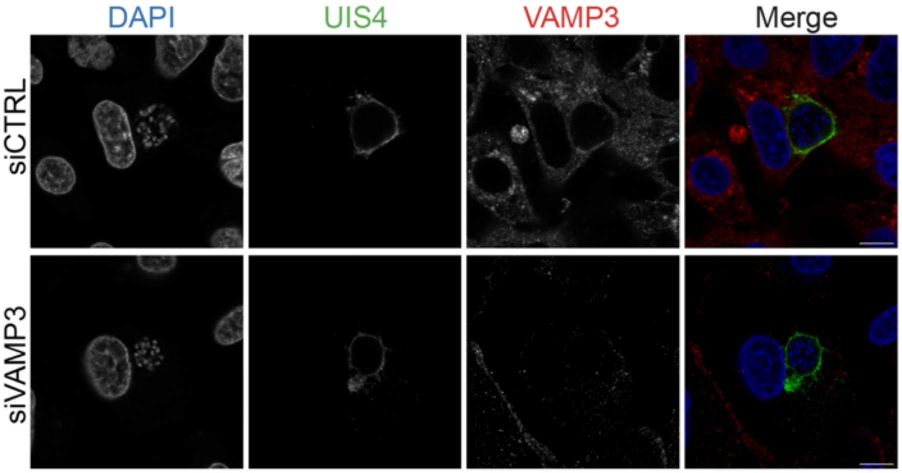

Figure S6. VAMP3 antibody specificity. HepG2 cells were reverse transfected with siCTRL or siVAMP3 (25 nM) for 48 h before infection with *P. berghei*-Luc. Cells were fixed at 48 hpi and stained for anti-PbUIS4 (green), anti-HsVAMP3 (red) and DAPI (blue). VAMP3 antibody does not stain the PVM in siVAMP3 treated cells. Scale bar is 10 µm.

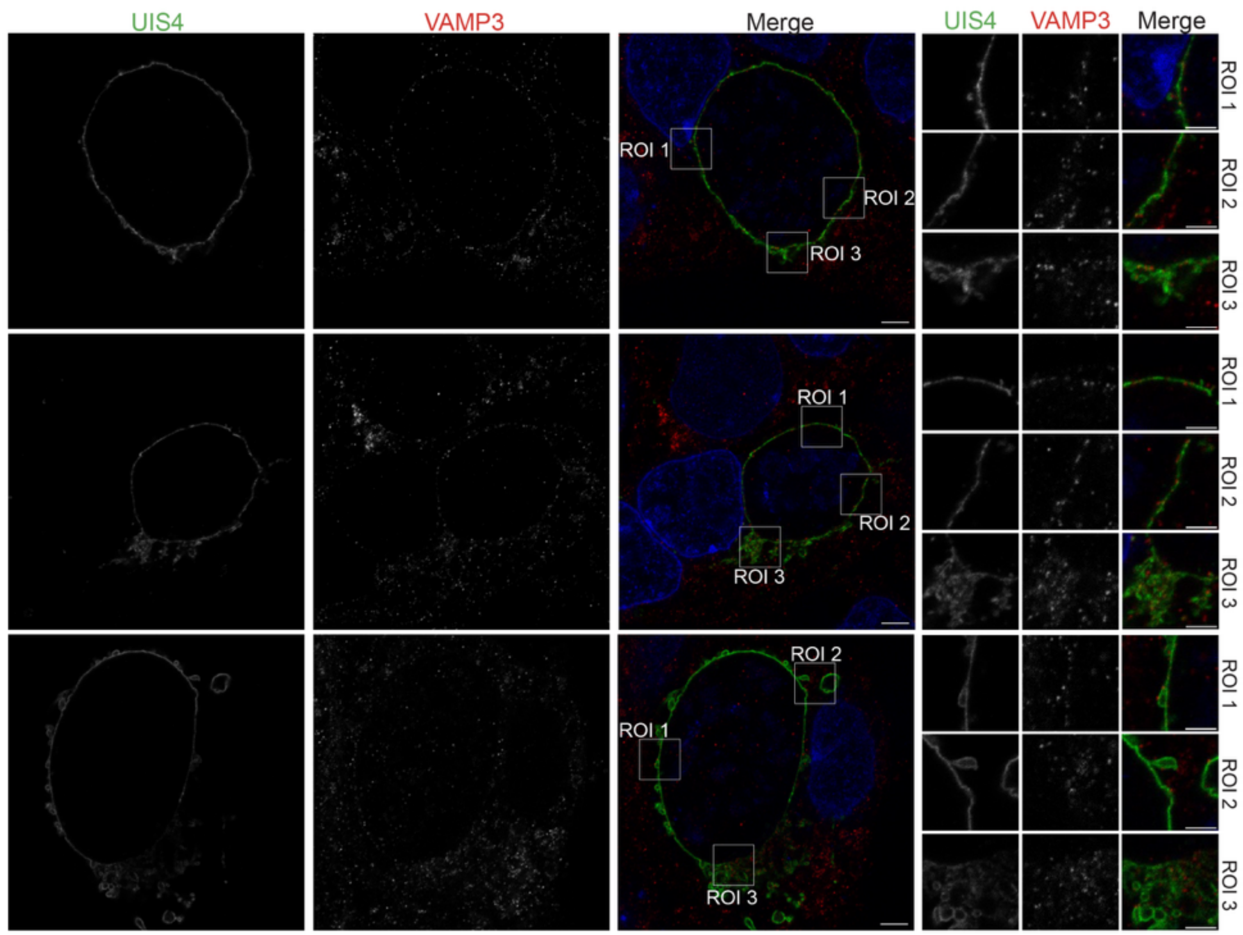

Figure S7. Expansion microscopy images of VAMP3 at the PVM. Additional confocal microscopy images of *P. berghei*-infected HepG2 cells fixed at 48 hpi and expanded 4.5-fold using an acrylamide hydrogel. Cells were stained with anti-PbUIS4 (green), anti-VAMP3 (red), and DAPI (blue). Scale bar is 10 μm and has not been adjusted for expansion. Insets of 3 regions of interest (ROI) of the PVM with scale bar of 5 μm, which has not been adjusted for expansion.

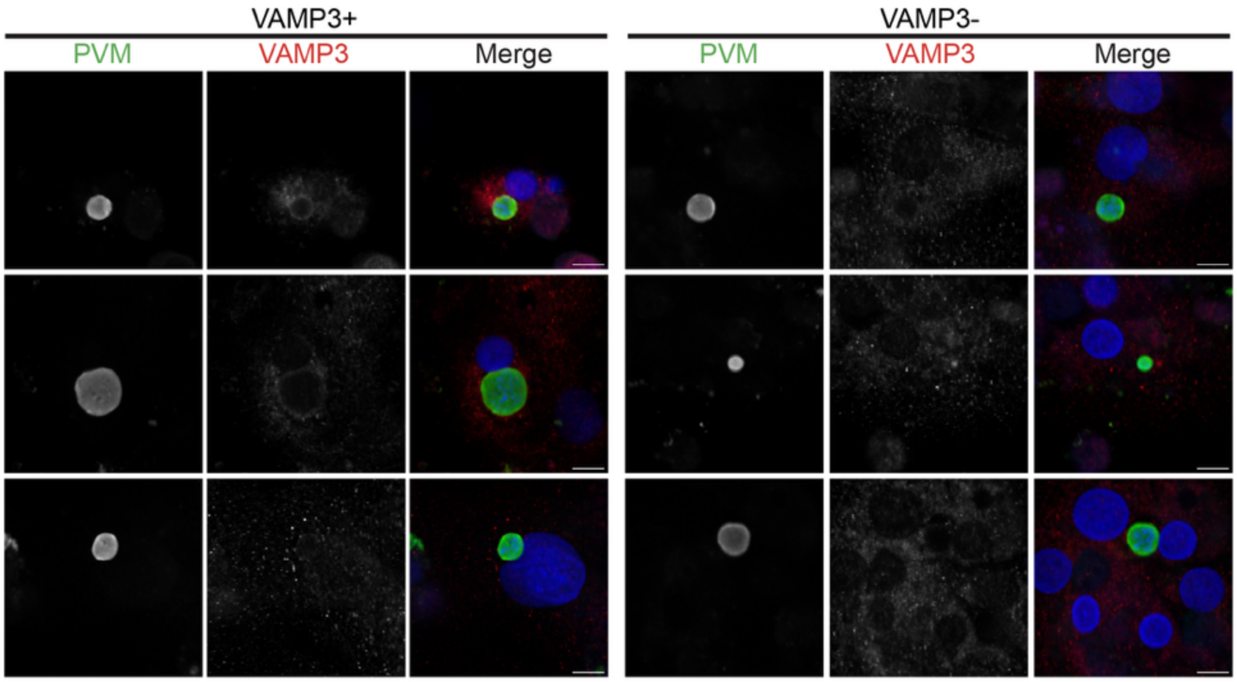

Figure S8. Images of VAMP3 association with *P. falciparum* LS. Additional confocal microscopy images of *P. falciparum*-infected PHHs fixed at 4 dpi. Cells were stained with anti-PbUIS4 (green), anti-HsVAMP3 (red) and DAPI (blue). Scale bar is 10 μm.

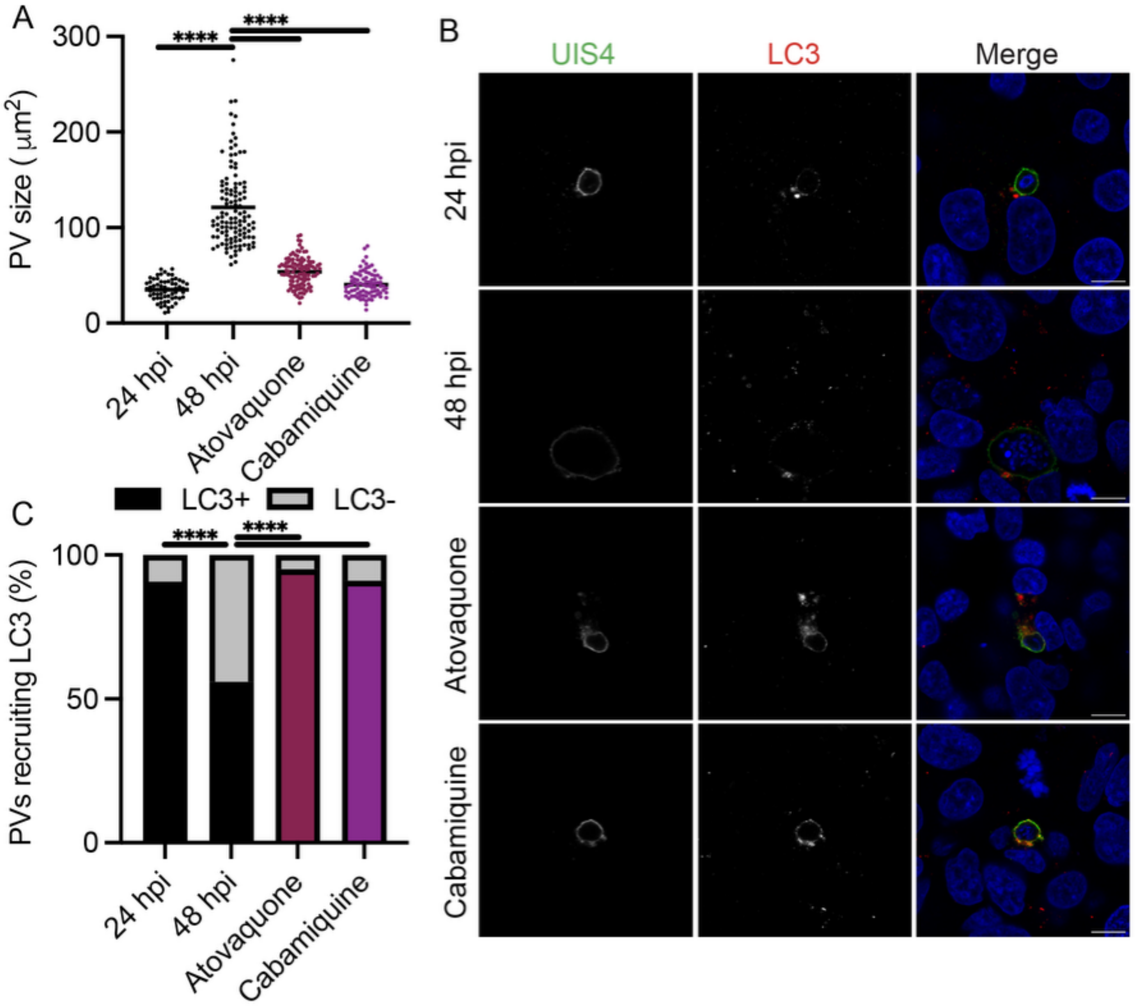

Figure S9. Treatment with atovaquone or cabamiquine reduces PV size and LC3 shedding. (A-C) HepG2 cells were infected with *P. berghei*-Luc and either fixed at 24 hpi or treated with DMSO, 20 nM atovaquone, or 10 nM cabamiquine from 24-48 hpi and fixed at 48 hpi. A) Cells were stained with anti-PbUIS4 and DAPI. PV size was determined by measuring area within UIS4 (Aivia 14). *n* = 3 biological replicates analyzing > 60 PVs. *p*-values display Welch's unpaired two-tailed t-test. B) Representative confocal microscopy images displaying PbUIS4 (green), HsLC3 (red) and DAPI (blue). Scale bar is 10 µm. C) PVs were assigned as positive for LC3 recruitment if there was substantial overlap between UIS4 and LC3 channels. *p*-values display Cochran-Armitage test. *n* = 3 biological replicates analyzing > 40 PVs. ****$p < 0.001$.

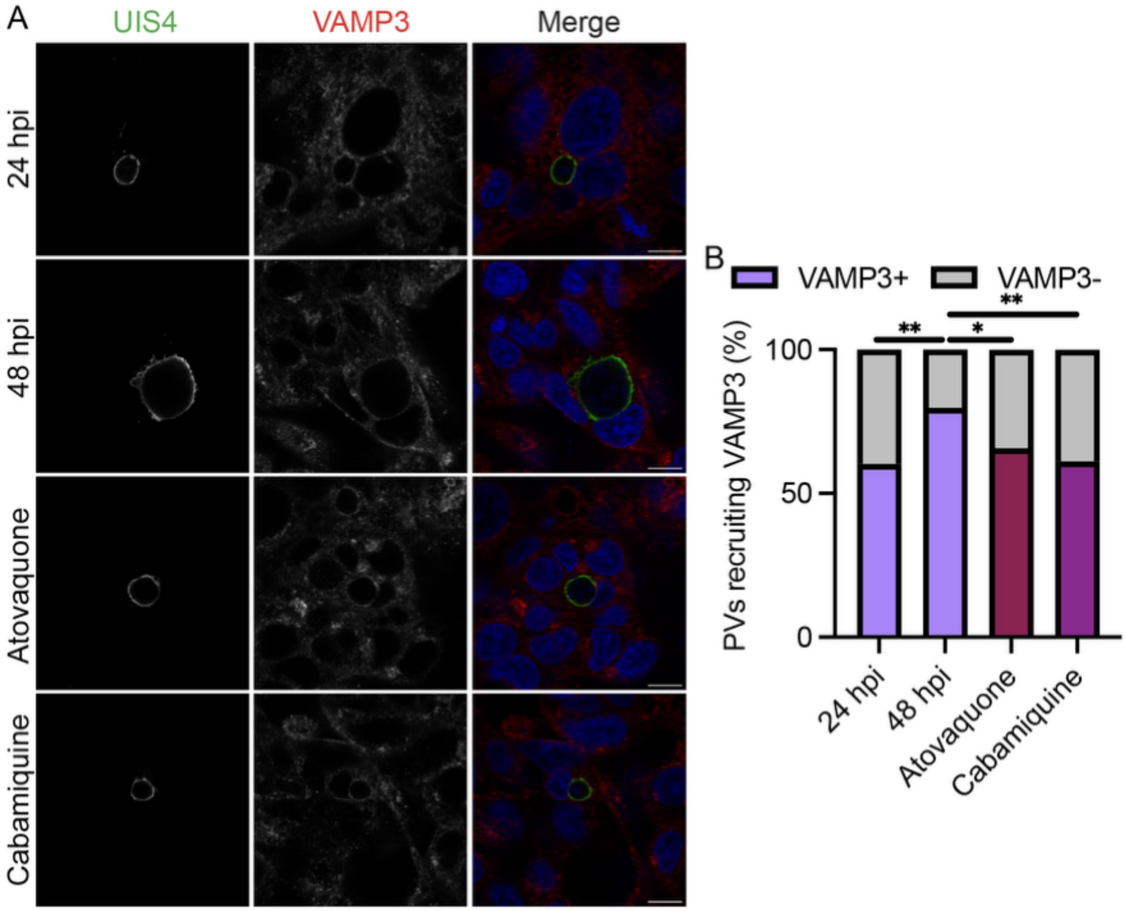

Figure S10. VAMP3 association decreases at the PVM with *P. berghei* inhibition. (A-B) HepG2 cells were infected with *P. berghei*-Luc and either fixed at 24 hpi or treated with DMSO, 20 nM atovaquone, or 10 nM cabamiquine from 24-48 hpi and fixed at 48 hpi. A) Representative confocal microscopy images displaying cells stained with anti-PbUIS4 (green), anti-HsVAMP3 (red), and DAPI (blue). Scale bar is 10 μm. B) PVs with > 1 μm^2 overlap in UIS4 and VAMP3 signal (Aivia 14 software) were assigned VAMP3 positive. *p*-values display Cochran-Armitage test. $n = 3$ biological replicates analyzing > 60 PVs. $^*p < 0.05$; $^{**}p < 0.01$.

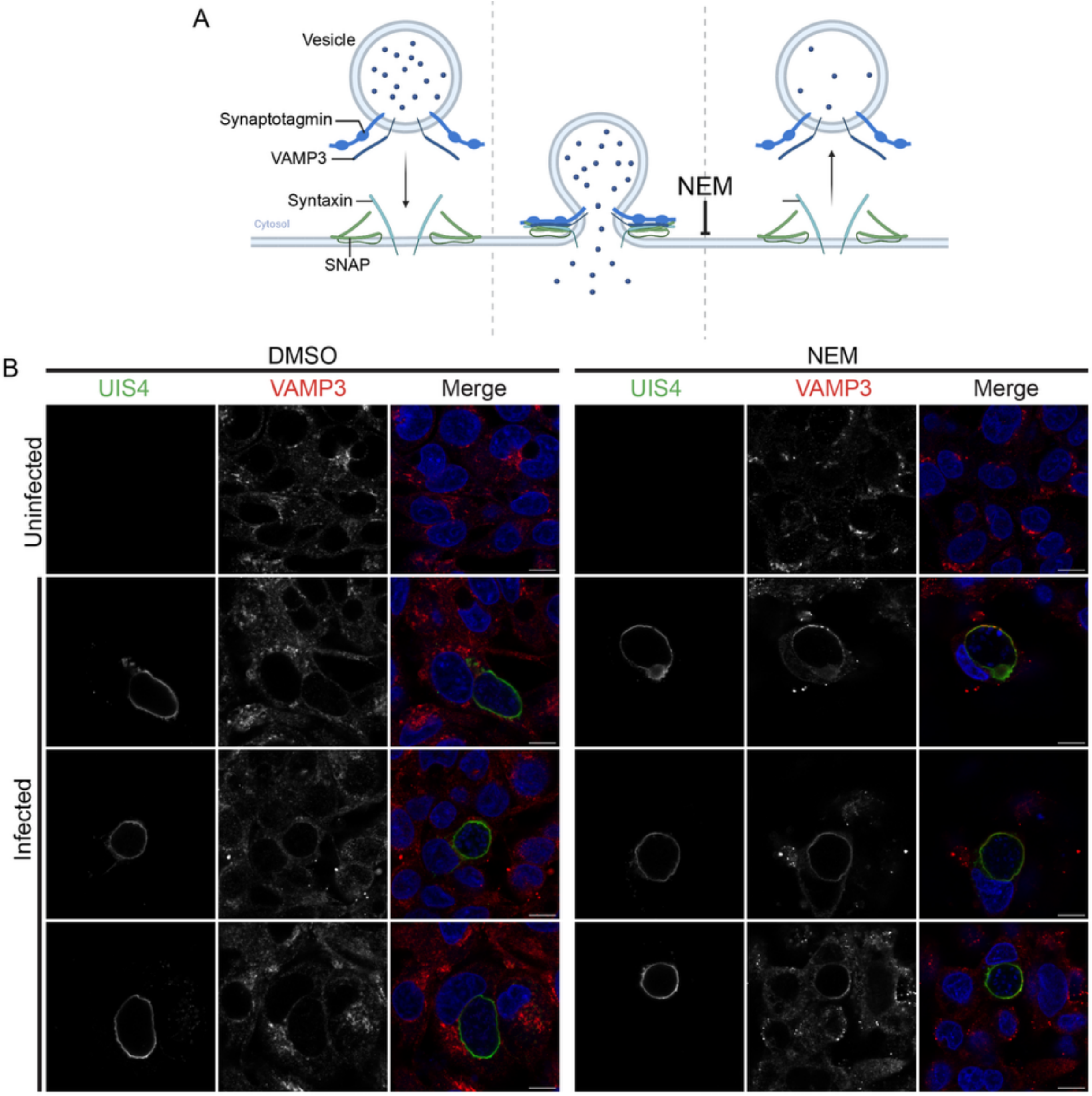

Figure S11. Confocal images showing VAMP3 association to *P. berghei* with DMSO or NEM treatment. A) Schematic showing NEM treatment blocks new vesicle fission, causing an accumulation of SNARE proteins on their target membranes. B) Additional confocal microscopy images of *P. berghei*-infected HepG2 cells. Cells were treated with DMSO or 250 μM NEM from 47.5-48 hpi and fixed at 48 hpi. Cells were stained with anti-PbUIS4 (green), anti-HsVAMP3 (red), and DAPI (blue). Scale bar is 10 μm.

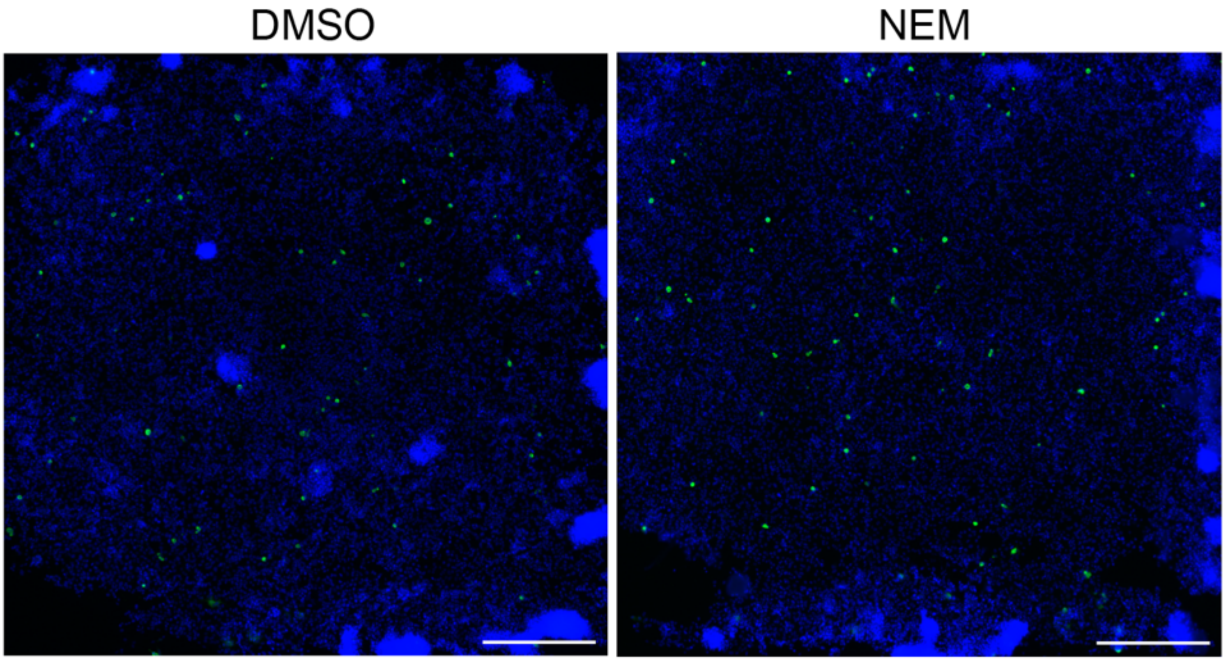

Figure S12. Visualization of *P. berghei* LS post NEM-treatment. Representative widefield images of an entire well of a 384-well plate of *P. berghei*-infected HepG2 cells. Cells were treated with DMSO or 250 μM NEM for 30 min at 47.5 hpi, and cells were fixed at 48 hpi. Cells were stained with DAPI (blue) and anti-PbUIS4 (green). No apparent hepatocyte or parasite death is observed with this treatment. Scale bar is 500 μm.

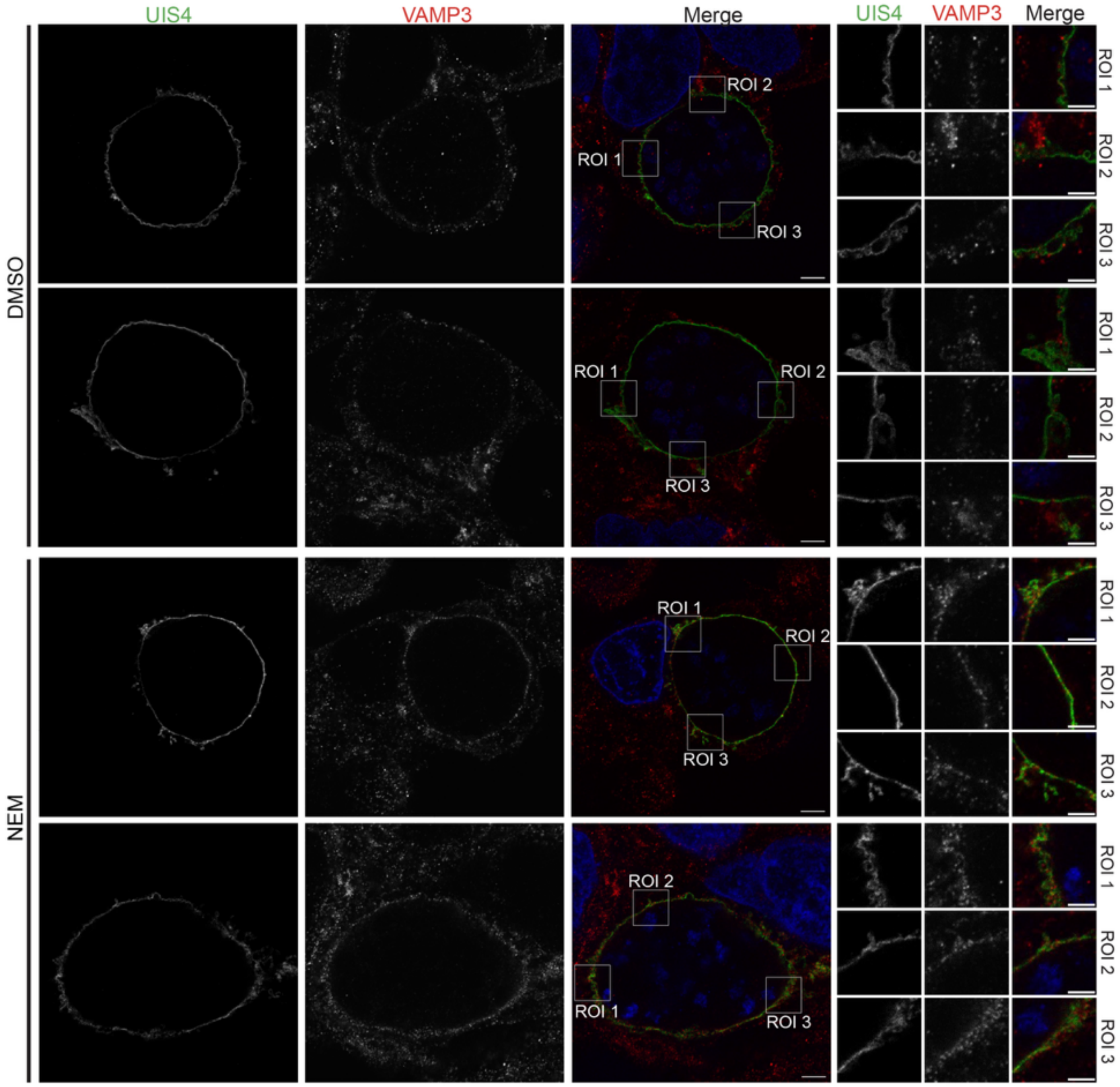

Figure S13. Expansion microscopy images of VAMP3 association after NEM treatment. Additional confocal microscopy images of *P. berghei*-infected HepG2 cells treated with DMSO or 250 µM NEM 47.5-48 hpi and fixed at 48 hpi. Cells were stained with anti-PbUIS4 (green), anti-VAMP3 (red), and DAPI (blue) and expanded 4.5-fold using an acrylamide hydrogel. Scale bar is 10 µm, not adjusted for expansion. Insets of 3 regions of interest (ROI) of the PVM with scale bar of 5 µm, not adjusted for expansion.

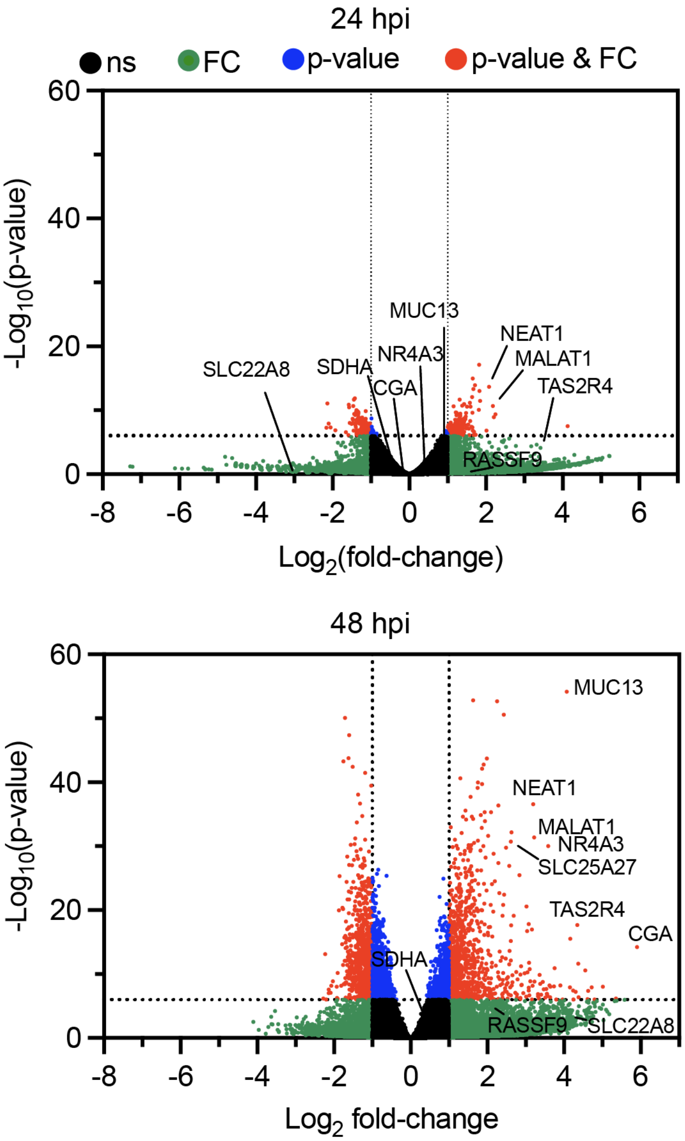

Figure S14. Long noncoding RNAs are upregulated during *P. berghei* infection. Volcano plots of differentially expressed genes at 24 and 48 hpi analyzing data from LaMonte et al. [9] Fold-change is reported on the *X* axis, and the *Y* axis shows $-\log_{10}$ adjusted *p*-value. Dashed lines on the *X* axis indicate a cutoff for $\log_2$ fold-change of > |1|, and dashed line on the *Y* axis indicate a *p*-value cutoff of $< 1\text{x}10^{-6}$. Green dots indicate genes above fold-change cutoff, blue dots show genes above the *p*-value cutoff, and red dots are genes above fold-change and *p*-value cutoff values. Long noncoding RNAs *MALAT1* and *NEAT1* indicated on plot.
